# Exposure to Environmental Chemicals from Environmental Tobacco Smoking in Korean Adolescents

**DOI:** 10.3390/toxics13070546

**Published:** 2025-06-29

**Authors:** Jung-Eum Lee, Ah-Reum Jo, Sunho Lee, Wanhyung Lee

**Affiliations:** 1 Department of Preventive Medicine, College of Medicine, Chung-Ang University, Seoul 06974, Republic of Korea; jexxxx@cau.ac.kr; 2 Department of Environmental Health Sciences, Graduate School of Public Health, Seoul National University, Seoul 08826, Republic of Korea; reum2692@snu.ac.kr; 3 Department of Pediatrics, CHA Ilsan Medical Center, CHA University, Goyang 13488, Republic of Korea; sunholee84@gmail.com

**Keywords:** environmental tobacco smoking, adolescents, environmental chemicals, exposure, epidemiology

## Abstract

**Background:** Environmental tobacco smoke (ETS) exposes adolescents to various environmental toxins, potentially affecting their developmental health. However, limited research exists on the associations between ETS exposure and the bodily burdens of environmental chemicals on adolescents. This study aimed to investigate the relationship between ETS exposure and the concentration of various environmental chemicals in adolescents, utilizing urinary cotinine as an objective biomarker. **Methods:** Data from 828 adolescents aged 12–17 years participating in the Korean National Environmental Health Survey (KoNEHS) were analyzed. ETS exposure was assessed via self-reported questionnaires and confirmed by urinary cotinine measurements. Levels of 33 environmental chemicals, including heavy metals, polycyclic aromatic hydrocarbons (PAHs), phthalates, phenols, volatile organic compounds (VOCs), and per- and polyfluoroalkyl substances (PFASs), were measured. Statistical analyses were conducted after adjusting for covariates. **Results:** Adolescents exposed to ETS showed significantly higher urinary cotinine and mono-(2-ethyl-5-carboxypentyl) phthalate (MECPP) concentrations than non-exposed adolescents. Additionally, significant positive correlations were observed between urinary cotinine levels and metabolites of PAHs (NAP, OHFlu), phenols (BPA, BPS), phthalates (MMP), and VOCs (t,t-MA) after adjustments. However, ETS exposure was not significantly associated with heavy metal concentrations. **Conclusions:** This study described the association between ETS exposure and environmental chemicals. A trend has been identified between ETS exposure in adolescents and increased bodily concentrations of various environmental chemicals, including PAHs, phenols, phthalates, and VOCs. As adolescence is a critical developmental period of vulnerability to environmental toxins, reducing ETS exposure to protect adolescents’ health and prevent potential lifelong health effects should be emphasized. This study was based on a cross-sectional design, and some confounding factors and measurement limitations may exist. Therefore, caution is needed in interpreting causality, and further research is recommended to determine more precise causality and long-term health effects.

## 1. Introduction

Environmental tobacco smoke (ETS), a significant source of indoor pollution, contains various toxic substances emitted directly from burning tobacco products [1]. Although the adverse health effects of ETS have been extensively documented in adults [2,3], limited research has specifically addressed its impact on adolescents. Recently, there have been studies analyzing the effects of ETS exposure, including electronic cigarettes as well as cigarettes, on the respiratory health of children and adolescents, but they did not secure statistical significance [4]. Adolescents represent a uniquely vulnerable population, as they typically lack autonomy in controlling exposure to ETS within their home or public environments [5]. Moreover, adolescence is a critical developmental stage during which exposure to environmental toxins could significantly influence lifelong health outcomes [6].

Previous studies have primarily focused on health effects related to ETS [7,8]; however, the relationship between ETS exposure and the burden of diverse environmental contaminants, including heavy metals, polycyclic aromatic hydrocarbons (PAHs), phthalates, phenols, and volatile organic compounds (VOCs), has rarely been investigated among adolescents. Given that tobacco combustion can generate and release various environmental chemicals, quantifying their accumulation in adolescent populations exposed to ETS is crucial for understanding potential health risks and informing adolescent health. The toxicological value of this study is the comprehensive analysis of ETS-related exposures to multiple environmental contaminants, using urinary cotinine as a quantitative biomarker of tobacco smoke exposure. Cotinine, a stable metabolite of nicotine, provides an objective and precise measure of ETS exposure levels [9].

This study hypothesizes that adolescents exposed to ETS have significantly higher bodily concentrations of environmental contaminants compared to non-exposed adolescents. To test this hypothesis, we analyzed representative data from the Korean National Environmental Health Survey (KoNEHS), focusing on urinary cotinine concentrations and the corresponding levels of 33 environmental chemicals, including heavy metals, polycyclic aromatic hydrocarbons (PAHs), phthalates, and per- and polyfluoroalkyl substances (PFASs). By investigating the association between exposure to ETS and the environmental contaminant status of adolescents, this study aims to demonstrate that ETS could be a prominent contributor to environmental contaminants. In this regard, it also aimed to provide scientific evidence to protect adolescents’ health from ETS.

## 2. Methods

### 2.1. Study Population

In this study, we analyzed data from adolescents (*n* = 828, middle and high school students) who participated in the KoNEHS cycle 4, conducted by the National Institute of Environmental Research (NIER) from 2018 to 2020. The Korean National Environmental Health Survey (KoNEHS) is a cross-sectional biomonitoring program held every three years since 2009 to assess exposure levels to major environmental chemicals in Korea. Using a sampling method, the KoNEHS cycle 4 provided representative data for middle and high school students nationwide, aged 12 to 17, with 67 schools selected as sampling sites.

The KoNEHS includes face-to-face interviews, physical examinations, biological sample collections, and questionnaires. Demographic and socioeconomic data along with factors related to exposure to environmental chemicals were collected through questionnaires and used as adjustment variables in the analysis. Urine and blood samples were occasionally collected from participants regardless of their fasting status. Among the 828 youth survey respondents, 26 who answered ‘currently smoke’ to the question on smoking were excluded from the analysis, leaving a final analysis target of 802 people.

### 2.2. Environmental Tobacco Smoking

Exposure to ETS was assessed using self-reported questionnaires. The questionnaire asked participants to answer questions about their smoking history and their exposure to second-hand smoke using a five-point scale (1: never; 2: 1–2 times per week; 3: 3–5 times per week; 4: 5–6 times per week; and 5: daily) [10]. Those who answered ‘none’ were classified into the control group, while those who answered ‘once a week or more’ were classified into the exposed group. The urinary cotinine concentration was used as an objective biomarker to quantify ETS exposure [11].

### 2.3. Environmental Chemical Analysis

Among the environmental chemicals in the KoNEHS cycle 4 data, 33 substances (excluding urine cotinine) were defined as environmental chemicals or metabolites. These environmental toxicants were categorized as follows: four types of heavy metals (blood lead, blood mercury, urine mercury, and urine cadmium), four types of PAHs (1-hydroxypyrene, 2-naphthol, 2-hydroxyfluorene, and 1-hydroxyphenanthrene), eight types of phthalate metabolites (mono-(2-ethyl-5-hydroxyhexyl) phthalate, mono-(2-ethyl-5-oxohexyl) phthalate, mono-n-butyl phthalate, mono-(2-ethyl-5-carboxypentyl) phthalate, monobenzyl phthalate, mono(3-carboxypropyl) phthalate, monoethyl phthalate, and monomethyl phthalate), nine types of environmental phenols (bisphenol A, bisphenol F, bisphenol S, triclosan, methylparaben, ethylparaben, propylparaben, butylparaben, and benzophenone-3), two types of VOC metabolites (trans,trans-muconic acid and benzylmercapturic acid), five types of PFASs (perfluorooctanoic acid, perfluorooctanesulfonate, perfluorohexanesulfonic acid, perfluorononanoic acid, and perfluorodecanoic acid), and one type of pyrethroid pesticide metabolite (3-phenoxybenzoic acid). The remaining substances were analyzed in the urine, except for two types of heavy metals (blood lead and blood mercury) and five types of PFASs (serum). Detailed descriptions of the analytical methods can be found in the Manual for the Analysis of Environmental Chemicals in Biological Samples from the National Environmental Health Survey Cycle 4 [12,13]. Because environmental chemical concentrations showed a right-skewed distribution, all values were log-transformed before analysis to improve normality and stabilize variance. Urine analytes were adjusted for urine creatinine concentration. All chemical analyses were conducted by certified laboratories following the QA/QC protocols, including inter-laboratory proficiency testing, the use of reference standards, and validation of analytical precision and accuracy. Detailed QA/QC performance metrics are provided in Supplementary S8.

### 2.4. Covariates

Age, sex, obesity level, economic status, and drinking habits were included as covariates. Obesity was categorized as underweight (body mass index, BMI less than 18.5 kg/m^2^), normal weight, or overweight (BMI greater than 25 kg/m^2^). Economic status was classified as high, medium, or low, based on self-reported survey responses. Drinking habits were categorized based on whether the individual consumed alcohol.

### 2.5. Statistical Analyses

The study population was divided into two groups based on their ETS exposure. The demographic characteristics of the exposed and non-exposed groups were analyzed using the χ^2^ test. Environmental chemical values were analyzed using the survey mean after accounting for stratification variables and survey sample weights, and differences according to ETS exposure were examined using Student’s *t*-test. Statistical significance was set at *p* < 0.05.

The proportion exceeding the 4th quartile of each environmental chemical value considering weight was compared according to ETS exposure. The correlations between urinary cotinine and other environmental chemical values were examined after adjusting for sex, age, socioeconomic status, BMI, and alcohol consumption. Additionally, the distribution trends of environmental chemical levels were analyzed based on urinary cotinine concentrations.

All data were log-transformed, and statistical analyses were performed using SAS (9.4; SAS Institute Inc., Cary, NC, USA) and R (version 4.2.1; R Foundation for Statistical Computing) software.

## 3. Results

### 3.1. Characteristics of the Study Population

Table 1 presents the characteristics of the 802 adolescents in grades 7–12 categorized by their ETS exposure. Significant differences were found between the ETS-exposed and non-ETS-exposed groups in terms of self-reported drinking (*p* < 0.0001) and smoking status (*p* < 0.0001), as well as household income (*p* = 0.010). Never-smokers comprised 623 (98.3%) and 155 individuals (92.3%) in the non-ETS-exposed and ETS-exposed groups, respectively. However, obesity did not differ significantly between the two groups.

### 3.2. Environmental Chemicals Associated with ETS

The concentrations of environmental chemicals are summarized in Table 2. The geometric mean of mono(2-ethyl-5-carboxypentyl) phthalate (MECPP), a metabolite of di-2-ethylhexyl phthalate (DEHP), was significantly higher in the ETS-exposed group (14.19 μg/g Cr) than in the non-ETS-exposed group (12.32 μg/g Cr) (*p* = 0.003). Moreover, urinary cotinine, a biomarker for ETS exposure, was found to be significantly elevated in the group exposed to ETS (*p* < 0.001). Although urinary t,t-muconic acid (tt-MA) levels increased slightly in the ETS-exposed group, the difference was not statistically significant (*p* = 0.074). No significant differences were observed in other chemical levels, including heavy metals, PAHs, phthalates, benzyl mercapturic acid (BMA), PFAS, or pesticides.

**Figure 1:**
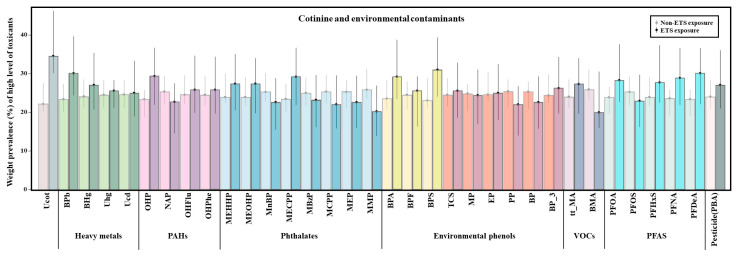
Weighted prevalence of high environmental chemical concentrations among study subjects by environmental tobacco smoking exposure group. Heavy metal: blood lead (BPb), blood mercury (BHg), urine mercury (Uhg), and urine cadmium (Ucd). PAHs (polycyclic aromatic hydrocarbons): 1-hydroxypyrene (OHP), 2-naphthol (NAP), 2-hydroxyfluorene (OHFlu), and 1-hydroxyphenanthrene (OHPhe). Phthalates: mono-(2-ethyl-5-hydroxyhexyl) phthalate (MEHHP), mono-(2-ethyl-5-oxohexyl) phthalate (MEOHP), mono-n-butyl phthalate (MnBP), mono-(2-ethyl-5-carboxypentyl) phthalate (MECPP), monobenzyl phthalate (MBzP), mono(3-carboxypropyl) phthalate (MCPP), monoethyl phthalate (MEP), and monomethyl phthalate (MMP). Environmental phenols: bisphenol A (BPA), bisphenol F (BPF), bisphenol S (BPS), triclosan (TCS), methyl paraben (MP), ethyl paraben (EP), propyl paraben (PP), butyl paraben (BP), and benzophenone-3 (BP_3). Nicotine: cotinine (COT). VOCs (volatile organic compounds): trans,trans-muconic acid (t,t-MA) and benzylmercapturic acid (BMA). PFASs (per- and polyfluoroalkyl substances): perfluorooctanoic acid (PFOA), perfluorooctanesulfonate (PFOS), perfluorohexanesulfonic acid (PFHxS), perfluorononanoic acid (PFNA), and perfluorodecanoic acid (PFDeA). Pesticide: 3-phenoxybenzoic acid (PBA).

Figure 1 illustrates the questionnaire-based weighted prevalence (%) of individuals with high exposure to environmental chemicals. High exposure was defined as levels above the 75th percentile for each chemical. Cotinine levels notably increased by 12.54%, indicating a marked increase in the prevalence of individuals in the high-exposure group following ETS. MECPP also increased by 5.79% in the ETS-exposed group. Moreover, an increasing trend (5% difference) was observed in concentrations of blood Pb, PFNA, PFDeA, and urinary OHP, BPA, and BPS in the ETS-exposed group. Several chemicals showed a slight decrease in the ETS group. These included 2-naphthol (NAP), the phthalate metabolites MnBP, MBzP, MCPP, MEP, methyl-, propyl-, and butyl parabens, and perfluorooctanesulfonate (PFOS). However, the prevalence of most differences was found to be less than 5%. Only BMA exhibited a notable decrease of 5.98%.

### 3.3. Environmental Chemicals Correlated with Urinary Cotinine Levels

The associations between environmental pollutants and cotinine levels generally remained consistent after adjusting for covariates, except that statistical significance was no longer observed for mono-(2-ethyl-5-oxohexyl) phthalate (MEOHP). Most substances displayed weak correlations with creatinine-adjusted urinary cotinine concentrations, with both Pearson and partial correlation coefficients showing |r| values below 0.15. Nonetheless, some correlations were statistically significant. Urinary metabolites of PAHs and VOCs, including naphthalene (NAP), 2-hydroxyfluorene (OHFlu), and t,t-MA, exhibited weak but significant positive correlations with cotinine (adjusted: NAP r = 0.090, *p* = 0.013; OHFlu r = 0.145, *p* < 0.0001; t,t-MA r = 0.126, *p* = 0.001). Among phthalates, all substances except monomethyl phthalate (MMP) showed negative correlations with cotinine; in the case of environmental phenols, bisphenol A (BPA) and bisphenol S (BPS) demonstrated weak but statistically significant positive correlations (adjusted: MMP: r = 0.094, *p* = 0.009; BPA: r = 0.082, *p* = 0.023; BPS: r = 0.098, *p* = 0.007). All PFAS compounds showed weak negative correlations with cotinine levels, with PFOS having the highest |r| value among them and showing strong statistical significance (adjusted *p* = 0.002, Table 3).

**Figure 2:**
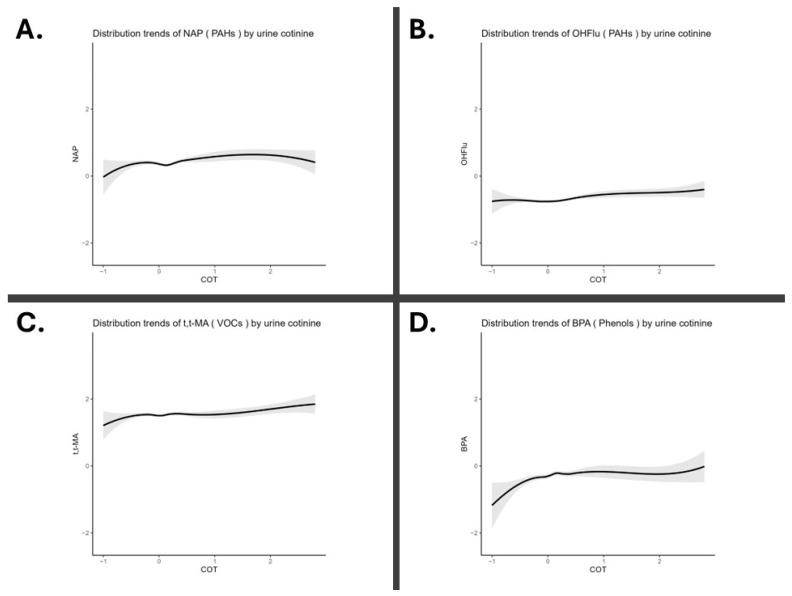
Distribution trends of environmental chemicals by urinary cotinine levels. All data were log-transformed. (**A**) PAHs (polycyclic aromatic hydrocarbons): 2-naphthol (NAP) (**B**) PAHs: 2-hydroxyfluorene (OHFlu). (**C**) VOCs (volatile organic compounds): trans,trans-muconic acid (t,t-MA). (**D**) Phenols: bisphenol A (BPA).

To explore the shape of the relationship between cotinine exposure and variations in environmental chemical concentrations (without covariate adjustments), locally weighted regression (LOESS) curves were modeled (Figure 2). The curves indicate a possible nonlinear relationship between cotinine and environmental chemical concentrations. For NAP and OHFlu, both types of PAHs, as well as for t,t-MA, a metabolite of VOCs, the Pearson correlations and LOESS trends showed similar upward patterns (Figure 2A–C). Furthermore, among environmental phenols, BPA displayed an increasing trend at lower cotinine concentrations, with a steeper slope at the highest concentration levels (Figure 2D). However, phthalates exhibited an initial increase at lower cotinine concentrations, followed by a decrease as cotinine levels rose (Supplementary Figure S3). Only results with substantial academic and research significance were retained, while less critical findings were presented in the supplementary materials.

## 4. Discussion

This study provides academic evidence demonstrating that adolescents exposed to ETS have significantly higher bodily concentrations of various environmental contaminants than the non-ETS group. Especially, using urinary cotinine as an objective biomarker of ETS exposure, we found clear associations between exposure to ETS and increased levels of PAHs, phthalates, phenols, and VOCs. These findings are particularly concerning for adolescent health, as this developmental period is characterized by rapid growth, hormonal regulation, and critical neurobehavioral development, all of which can be adversely affected by environmental contaminants [14]. Even low-level ETS exposure poses potential long-term health risks, underscoring the importance of reducing adolescent exposure to ETS.

ETS has been widely recognized as a critical public health concern due to its diverse range of adverse health effects. ETS exposure has been robustly associated with increased risks of respiratory disorders such as asthma and chronic bronchitis, cardiovascular diseases including hypertension and ischemic heart disease, various forms of cancer, and neurodevelopmental impairments [15]. Furthermore, ETS contains numerous toxic compounds, including PAHs, VOCs, and endocrine-disrupting chemicals such as phthalates and phenols, which can profoundly influence adolescent development and health outcomes [16]. Considering the immunotoxic potential of many chemicals identified in our study, adolescents may face compounded risks of immune dysfunction from ETS exposure, further emphasizing the importance of targeted public health interventions.

In this study, urinary PAH metabolites such as NAP and OHFlu showed significant positive correlations with urinary cotinine levels, indicating a dose–response relationship with ETS exposure. However, no statistically significant differences in PAH metabolite concentrations were directly observed between the ETS-exposed and non-exposed groups. This apparent discrepancy may be due to the frequency, intensity, or the limitations of self-report-based ETS exposure classification, which is also subject to recall errors and underreporting. Previous research has clearly demonstrated elevated urinary PAH metabolite concentrations among actively smoking adolescents, or those heavily exposed to ETS environments [17,18]. Biologically, PAHs are well known to exert harmful health effects through multiple mechanisms, including oxidative stress induction, inflammatory responses, endocrine disruption, and genotoxicity [19]. In addition, previous epidemiological studies have reported that when urinary NAP concentrations exceed 1.60 μg/L, the prevalence of asthma is more than three times higher [20]. Adolescence is a critical developmental period with increased sensitivity to environmental stress, and even small increases in PAH metabolites associated with environmental tobacco smoke (ETS) exposure may have negative effects on adolescent health. Therefore, since there are no precise reference values for PAHs, future studies utilizing more sensitive exposure assessment methodologies or longitudinal studies would be helpful in elucidating the precise relationship between low-level ETS exposure and PAH metabolite accumulation in adolescents.

Urine t,t-MA, a well-established metabolite of benzene, showed a significant positive correlation with urinary cotinine levels in our study. This finding suggests a clear association between ETS exposure and internal benzene burden in adolescents. Benzene is a volatile organic compound emitted during the incomplete combustion of tobacco, and t,t-MA has been widely used as a biomarker of low-level benzene exposure in both occupational and general environmental health studies [21]. Given the short biological half-life of t,t-MA, the observed association underscores the relevance of recent or ongoing ETS exposure in contributing to the internal benzene dose among adolescents. In adolescents, higher urinary t,t-MA levels have been associated with measurable deficits in sustained attention, reduced working memory, and even early metabolic disturbances such as insulin resistance, potentially mediated through oxidative stress pathways [22,23]. These findings reinforce the argument that even low-level ETS exposure can have biologically meaningful and developmentally disruptive consequences. Given that benzene is a genotoxic carcinogen with no safe exposure limit [24], the association between ETS-related urinary cotinine and t-MA in adolescents highlights the urgent need for preventive interventions, particularly during a developmental period of increased vulnerability.

Our study identified significant positive correlations between urinary cotinine and BPA and BPS concentrations in adolescents. While bisphenols are not conventionally recognized as major constituents of tobacco smoke, recent evidence suggests that ETS may serve as an indirect source of exposure through complex secondary mechanisms. Previous studies have shown that filters may contain BPA as a plasticizing additive [25]. Adolescents who live with parents who smoke may be exposed to BPA through thirdhand smoke or direct contact when tobacco-related substances are deposited on indoor surfaces, such as on smokers’ hands [26]. These residues can also contaminate household dust, which can be inhaled or ingested, especially in younger individuals who exhibit more frequent hand-to-mouth behavior. Our findings are consistent with reports from the Flemish Environment and Health Study (FLEHS), which demonstrated higher BPA and BPF levels in adolescents exposed to passive smoking [27]. These findings suggest that ETS-exposed environments may act as reservoirs for a broader set of environmental toxicants, not limited to classical tobacco combustion by-products. From a toxicological perspective, bisphenols are endocrine-disrupting chemicals (EDCs) that interfere with estrogen, androgen, and thyroid hormone signaling. Adolescents are particularly vulnerable to EDCs due to ongoing endocrine maturation and neurodevelopment. BPA and BPS exposure during adolescence has been associated with early puberty, altered body composition, and increased risk of obesity and insulin resistance [28]. Thus, even modest elevations in bisphenol levels among ETS-exposed adolescents may have significant implications for hormonal and metabolic health trajectories. These findings highlight the importance of considering ETS not only as a direct toxicant, but also as a proxy for broader environmental exposures in shared indoor environments.

In our study, associations between ETS exposure and urinary phthalate metabolite concentrations were generally inconsistent. While MECPP was significantly elevated in the ETS-exposed group, other metabolites showed no clear pattern, and some (e.g., MnBP, MBzP) even tended to be lower in this group. These findings mirror the previous literature reporting mixed associations between tobacco smoke exposure and urinary phthalates in adolescents and adults [29,30]. The heterogeneity of these findings may reflect the fact that phthalates are not primary constituents of tobacco smoke itself. Rather, they are ubiquitous in modern environments—used in food packaging, personal care products, vinyl flooring, adhesives, and many consumer goods. As such, elevated phthalate levels in ETS-exposed adolescents may reflect broader lifestyle or environmental differences rather than direct chemical uptake via smoke inhalation [31]. Biologically, phthalates are well-established endocrine-disrupting chemicals, with documented associations with altered puberty timing, reproductive hormone changes, and neurodevelopmental impacts in children and adolescents [32]. Therefore, urinary phthalate levels in ETS-exposed adolescents should be interpreted as possible indicators of compound environmental vulnerabilities, rather than as direct consequences of tobacco smoke alone.

In this study, urinary cotinine levels were negatively correlated with serum concentrations of several PFAS compounds, including PFOA, PFOS, PFHxS, PFNA, and PFDeA. This inverse association may initially appear counterintuitive, as it suggests that adolescents with higher ETS exposure may have lower PFAS body burdens. However, similar trends have been reported in previous studies [33]. The underlying mechanisms for this relationship remain unclear and may involve a combination of biological, behavioral, and sociodemographic factors. Biologically, PFASs are known for their long biological half-lives and persistent accumulation in serum, in contrast to ETS-related metabolites such as cotinine, which reflect short-term exposures [34]. Thus, cotinine and PFAS levels may represent temporally distinct exposure patterns, making direct correlation difficult. Additionally, it has been hypothesized that higher urinary cotinine concentrations could be associated with altered renal function or metabolic pathways that influence PFAS elimination kinetics, though evidence on this remains limited and inconclusive. Given the complex and sometimes contradictory findings in the literature on the relationship between ETS and PFASs warrants further investigation using longitudinal data and more detailed covariate control, including socioeconomic status, diet, and consumer product use.

In our study, we found no significant association between urinary cotinine and pyrethroid. This finding suggests that ETS may not be a primary contributor to pyrethroid exposure in adolescents. However, previous studies have produced mixed results. For example, some research has reported higher urinary PBA levels among active smokers compared to non-smokers, potentially reflecting pyrethroid residues on tobacco leaves or transfer through hand-to-mouth activity during cigarette handling [35]. In contrast, others have found no clear association [36]. Pyrethroid exposure in the general population is primarily attributed to the use of household insecticides, agricultural pesticide residues on food, and contaminated indoor dust. While it is plausible that tobacco users may be incidentally exposed through pyrethroid-treated tobacco leaves or via dermal contact with pesticide-contaminated surfaces [37], these pathways are likely minor compared to more direct routes such as dietary ingestion or residential spraying. Given the limited scope of the exposure pathways examined in this analysis, future studies should incorporate more comprehensive data on insecticide use within households, dietary intake of pesticide residues, and frequency of contact with treated surfaces. This would allow for better attribution of pyrethroid exposure sources and to clarify whether ETS plays any contributory role in specific subpopulations.

Contrary to expectations, this study found no significant associations between ETS and heavy metal exposure. This could be attributed to the relatively low levels of heavy metals introduced through ETS, which may be insufficient for detection [38]. In a study on adults, oral exposure to heavy metals was the most common route, with food intake accounting for approximately 70%. Therefore, it is thought that the non-smoking route played a more dominant role in heavy metal accumulation in adolescence [39]. In addition, adolescents have a relatively shorter exposure period and limited bioaccumulation time compared to adults, so differences in body burden due to ETS exposure may not appear. Therefore, follow-up studies are needed to precisely identify differences in heavy metal concentrations in the body by exposure source and route, such as air, food, and drinking water.

In our analysis, the biomarker levels of environmental chemicals among adolescents exposed and not exposed to ETS showed varied concentrations. When evaluating these levels relative to adults in the Korean general population, as well as adolescents and adults reported in international Human Biomonitoring (HBM) programs, several characteristics emerged. Generally, the biomarker concentrations in Korean adolescents observed in our study were comparable or slightly lower than those previously reported in Korean adults (KoNEHS adult data), and were broadly consistent with the levels reported in adolescents in other countries’ HBM programs, such as the U.S. NHANES and European HBM4EU [40,41]. For example, urinary concentrations of phthalates (e.g., MECPP) and phenols (e.g., BPA) in our adolescent population were similar to or slightly below those reported in Korean adults and adolescents from Western countries [41]. This similarity may reflect the widespread use of consumer products containing these chemicals across populations, regardless of age. One notable characteristic of our adolescent study population is their potentially lower cumulative lifetime exposure compared to adults, which may partly explain somewhat lower or comparable biomarker levels relative to adult populations. Additionally, the observed chemical concentrations in Korean adolescents might reflect distinct cultural and behavioral patterns, such as differences in dietary habits, lifestyle, product usage, and indoor environments. Moreover, due to Korea’s unique indoor and dietary culture, including high rice and seafood consumption (leading to potential elevated metal exposure) and specific consumer product usage patterns, our findings could reflect population-specific chemical exposure profiles. Despite the lack of significant ETS-driven elevation in heavy metal levels, this cultural context is important for interpreting biomonitoring data within this population. Given these considerations, further comparative analyses incorporating detailed exposure assessments, dietary and behavioral surveys, and international biomonitoring data could provide additional insight into the unique exposure characteristics of Korean adolescents and help to better contextualize ETS-related chemical exposure within a broader public health framework.

The findings from our study highlight the need for stronger public health measures targeting ETS exposure among adolescents. To effectively mitigate the identified risks, policymakers should consider implementing or reinforcing several strategies. First, stricter indoor smoking bans, especially in residential buildings, schools, and public facilities frequented by adolescents, are critical. Previous studies demonstrated that comprehensive smoke-free legislation significantly reduced ETS exposure levels and associated adverse health outcomes in adolescents [42,43]. Second, educational programs specifically tailored to adolescents and their caregivers could enhance awareness about the risks associated with ETS and promote healthier indoor environments. Such educational initiatives should be incorporated into school curricula and community health programs to ensure sustained behavioral changes. Lastly, enforcing regular monitoring and reporting of ETS exposure levels among adolescents through national surveys like KoNEHS can provide critical data to assess the effectiveness of implemented policies and guide continuous improvement in public health interventions.

This study has several strengths. First, it uniquely focuses on adolescents, a population that is particularly vulnerable to environmental exposures due to ongoing physical growth, hormonal changes, and neurological development. Second, by employing urinary cotinine as a quantitative biomarker of ETS exposure, this research provides objective and reliable evidence linking ETS to the accumulation of multiple environmental chemicals. Finally, utilizing data from the KoNEHS ensures nationally representative findings, enhancing the generalizability and relevance of the results to adolescent populations broadly.

This study has several limitations. First, while this study identified significant associations between ETS exposure and several environmental chemicals, it is important to recognize that the mechanisms by which adolescents accumulate these chemicals are likely to be multifactorial and extend beyond direct inhalation. In addition to side stream smoke inhalation, non-smoking youth can be exposed via secondary pathways, such as semi-volatile organic compounds (SVOCs) re-emitted into indoor air, dermal contact with smoke residues, and thirdhand smoke from contaminated surfaces and dust [44,45]. Although certain chemicals such as bisphenols and VOC metabolites (e.g., t,t-MA) were significantly correlated with ETS, these substances are not direct components of tobacco smoke. Their elevated concentrations among adolescents exposed to ETS may indicate secondary exposure mechanisms, including thirdhand smoke residues, altered indoor environments, or behaviors typical in smoking households. Therefore, the associations identified likely reflect broader environmental and behavioral contexts rather than direct ETS inhalation alone. For several of the chemicals analyzed in this study, including phthalates, phenols, PFASs, and heavy metals, ETS was not the primary source of exposure. For example, cadmium and mercury are primarily ingested through rice and fish consumption [46], or phthalates and bisphenols through personal care products and food packaging [31], and for PFASs, through contaminated indoor environments or drinking water [47]. Furthermore, youth exposed to ETS may be exposed to other hazardous chemicals depending on their living environment and behavior. For example, one study found that reducing ventilation after quitting indoor smoking may increase concentrations of non-tobacco-related indoor pollutants [48]. We recognize that additional exposure sources could be a confounding factor, particularly with regard to chemicals such as MECPP, which predominantly originate from dietary and household dust rather than tobacco smoke. The elevated MECPP levels observed in adolescents exposed to ETS may therefore represent broader environmental differences associated with households where people smoke, including dietary patterns, consumer product usage, and indoor dust levels [49]. Future studies should aim to account for these environmental and behavioral factors comprehensively to clarify their respective contributions to the observed associations. Thus, the elevated concentrations observed among ETS-exposed adolescents may partially reflect differences in unmeasured social, behavioral, and environmental characteristics. For example, households that permit indoor smoking may also engage in different dietary practices, use different consumer products, or have lower environmental health literacy, all of which could indirectly affect chemical body burden [50]. Second, while our models adjusted for key sociodemographic covariates including household income, age, sex, BMI, and alcohol consumption, we acknowledge that critical confounding variables, particularly dietary habits and consumer product usage, were not available in the dataset and therefore not controlled for in this analysis. Urinary cotinine levels not only quantify direct exposure to tobacco smoke, but may also indirectly capture adolescents’ cumulative burden of environmental chemical exposures, mediated through shared social and environmental determinants. Recognizing ETS as a multidimensional risk marker, particularly during this sensitive developmental period, reinforces the need for comprehensive strategies to mitigate adolescent exposure to both tobacco smoke and the broader spectrum of harmful environmental chemicals associated with it. Third, the cross-sectional design precludes the ability to infer temporal or causal relationships between ETS exposure and the accumulation of environmental chemicals. Longitudinal follow-up studies are warranted to evaluate the trajectory of chemical accumulation over time and to investigate associated health outcomes during adolescence and into adulthood. Finally, although the study benefits from a nationally representative sample, the number of participants in the ETS-exposed group (n = 168) may be insufficient for robust subgroup or dose–response analyses. The limited sample size may reduce statistical power and increase the risk of type II error. Future research should consider larger cohorts and pooled multi-cycle datasets to allow for more detailed stratified analyses and improve external validity.

## 5. Conclusions

This study described the association between ETS exposure and environmental chemicals in adolescents. A trend has been identified between ETS exposure in adolescents and increased bodily concentrations of various environmental chemicals, including PAHs, phenols, phthalates, and VOCs. This study emphasizes the urgent need for targeted public health interventions to reduce ETS exposure among adolescents. Practical measures, such as stricter enforcement of indoor smoking bans, adolescent-specific educational campaigns, and ongoing nationwide biomonitoring programs, are necessary to effectively reduce the exposure burden and protect adolescent health.

## Figures and Tables

**Table 1:** Distribution of the study population by exposure to environmental tobacco smoking.

		Exposure to Environmental Tobacco Smoking		*p*-Value *
		No	Yes	
Total		634 (79.1)	168 (20.9)	
Gender	Male	284 (44.8)	84 (50.0)	0.258
	Female	350 (55.2)	84 (50.0)	
School year	7th	106 (16.7)	19 (11.3)	0.456
	8th	95 (15.0)	24 (14.3)	
	9th	102 (16.1)	25 (14.9)	
	10th	114 (18.0)	31 (18.5)	
	11th	123 (19.4)	32 (19.0)	
	12th	94 (14.8)	37 (22.0)	
Drinking	No	447 (70.5)	95 (56.5)	<0.0001
	Yes	187 (29.5)	73 (43.5)	
Smoking	Never	623 (98.3)	155 (92.3)	<0.0001
	Ex-smoker	11 (1.7)	13 (7.7)	
Income ^a^ ($/month)	≤1520	34 (5.4)	18 (10.7)	0.010
	1520–3800	280 (44.1)	87 (51.8)	
	3800≤	285 (45.0)	54 (32.1)	
	No response	35 (5.5)	9 (5.4)	
Obesity ^b^ (kg/m^2^)	Underweight	86 (13.6)	23 (13.7)	0.782
	Normal weight	428 (67.5)	108 (64.3)	
	Overweight	120 (18.9)	37 (22.0)	

* Values are presented as numbers (%). *p*-values were calculated using the chi-square test. ^a^ Average monthly income over the past year and the exchange rate of 1315 won per dollar was applied. ^b^ Obesity calculated as weight (kg)/height^2^ (m^2^). Underweight < 18.5 kg/m^2^, 18.5 kg/m^2^ ≤ Normal weight < 25 kg/m^2^, and 25 kg/m^2^ ≤ Overweight.

**Table 2:** The geometric mean concentration of environmental chemicals by exposure to environmental tobacco smoking.

Environmental Chemicals		Environmental Tobacco Smoking Exposure				*p*-Value **
		No		Yes		
		n	GM (SE) *	n	GM (SE)	
Nicotine ^a^	COT (μg/g cr.)	625	1.34 (1.05)	166	1.81 (1.11)	<0.001
Heavy metal ^b^	BPb (μg/dL)	631	0.80 (1.02)	168	0.84 (1.05)	0.318
	BHg (μg/L)	631	1.38 (1.02)	168	1.36 (1.05)	0.687
	Uhg (μg/g cr.)	630	0.18 (1.03)	166	0.18 (1.05)	0.866
	Ucd (μg/g cr.)	630	0.08 (1.07)	166	0.08 (1.12)	0.616
PAHs ^c^	OHP (μg/g cr.)	618	0.06 (1.06)	165	0.07 (1.09)	0.187
	NAP (μg/g cr.)	618	2.37 (1.06)	165	2.35 (1.08)	0.824
	OHFlu (μg/g cr.)	618	0.19 (1.06)	165	0.19 (1.07)	0.456
	OHPhe (μg/g cr.)	618	0.04 (1.05)	165	0.05 (1.10)	0.309
Phthalates ^d^	MEHHP (μg/g cr.)	628	6.85 (1.12)	166	6.67 (1.20)	0.994
	MEOHP (μg/g cr.)	628	3.76 (1.12)	166	3.74 (1.20)	0.829
	MnBP (μg/g cr.)	628	12.08 (1.14)	166	10.95 (1.20)	0.667
	MECPP (μg/g cr.)	628	12.32 (1.04)	166	14.19 (1.05)	0.003
	MBzP (μg/g cr.)	628	0.45 (1.12)	166	0.46 (1.17)	0.457
	MCPP (μg/g cr.)	628	0.16 (1.04)	166	0.16 (1.07)	0.855
	MEP (μg/g cr.)	628	4.28 (1.11)	166	4.07 (1.16)	0.556
	MMP (μg/g cr.)	628	2.27 (1.07)	166	2.04 (1.09)	0.249
Environmental phenols ^e^	BPA (μg/g cr.)	630	0.53 (1.08)	166	0.60 (1.11)	0.192
	BPF (μg/g cr.)	630	0.16 (1.16)	166	0.16 (1.25)	0.533
	BPS (μg/g cr.)	630	0.08 (1.09)	166	0.09 (1.15)	0.109
	TCS (μg/g cr.)	630	0.12 (1.06)	166	0.11 (1.07)	0.121
	MP (μg/g cr.)	630	7.90 (1.08)	166	8.31 (1.13)	0.820
	EP (μg/g cr.)	630	32.72 (1.12)	166	33.32 (1.17)	0.971
	PP (μg/g cr.)	630	0.38 (1.10)	166	0.35 (1.18)	0.848
	BP (μg/g cr.)	630	0.45 (1.04)	166	0.46 (1.06)	0.740
	BP_3 (μg/g cr.)	630	0.55 (1.08)	166	0.50 (1.14)	0.417
VOCs ^f^	t,t-MA (μg/g cr.)	610	33.75 (1.06)	163	37.15 (1.07)	0.074
	BMA (μg/g cr.)	610	2.97 (1.04)	163	2.83 (1.06)	0.105
PFAS ^g^	PFOA (μg/L)	631	3.62 (1.03)	168	3.77 (1.05)	0.505
	PFOS (μg/L)	631	7.92 (1.04)	168	8.05 (1.06)	0.866
	PFHxS (μg/L)	631	2.54 (1.07)	168	2.51 (1.14)	0.972
	PFNA (μg/L)	631	0.91 (1.03)	168	0.95 (1.05)	0.194
	PFDeA (μg/L)	631	0.44 (1.02)	168	0.47 (1.04)	0.169
Pesticide ^h^	PBA	617	0.37 (1.06)	164	0.41 (1.13)	0.274

* Values are presented as geometric means (GMs), and standard errors (SEs). ** The *p*-value was calculated using Student’s *t*-test. ^a^ Nicotine: cotinine (COT). ^b^ Heavy metal: blood lead (BPb), blood mercury (BHg), urine mercury (Uhg), and urine cadmium (Ucd). ^c^ PAHs (polycyclic aromatic hydrocarbons): 1-hydroxypyrene (OHP), 2-naphthol (NAP), 2-hydroxyfluorene (OHFlu), and 1-hydroxyphenanthrene (OHPhe). ^d^ Phthalates: mono-(2-ethyl-5-hydroxyhexyl) phthalate (MEHHP), mono-(2-ethyl-5-oxohexyl) phthalate (MEOHP), mono-n-butyl phthalate (MnBP), mono-(2-ethyl-5-carboxypentyl) phthalate (MECPP), monobenzyl phthalate (MBzP), mono(3-carboxypropyl) phthalate (MCPP), monoethyl phthalate (MEP), and monomethyl phthalate (MMP). ^e^ Environmental phenols: bisphenol A (BPA), bisphenol F (BPF), bisphenol S (BPS), triclosan (TCS), methyl paraben (MP), ethyl paraben (EP), propyl paraben (PP), butyl paraben (BP), and benzophenone-3 (BP_3). ^f^ VOCs: trans,trans-muconic acid (t,t-MA) and benzylmercapturic acid (BMA). ^g^ PFASs (per- and polyfluoroalkyl substances): perfluorooctanoic acid (PFOA), perfluorooctanesulfonate (PFOS), perfluorohexanesulfonic acid (PFHxS), perfluorononanoic acid (PFNA), and perfluorodecanoic acid (PFDeA). ^h^ Pesticide: 3-phenoxybenzoic acid (PBA).

**Table 3:** Correlation between urinary cotinine and environmental chemicals.

Environmental Chemicals		Crude		Adjusted **	
		*r*	*p*-Value *	*r*	*p*-Value *
Heavy metal ^a^	BPb	0.051	0.155	0.023	0.521
	BHg	0.031	0.387	0.010	0.790
	Uhg	0.008	0.832	0.005	0.889
	Ucd	0.000	0.996	0.006	0.879
PAHs ^b^	OHP	0.043	0.232	0.040	0.271
	NAP	0.100	0.005	0.090	0.013
	OHFlu	0.135	<0.001	0.145	<0.0001
	OHPhe	0.061	0.087	0.063	0.083
Phthalates ^c^	MEHHP	−0.105	0.003	−0.087	0.016
	MEOHP	−0.072	0.043	−0.055	0.127
	MnBP	−0.092	0.010	−0.073	0.044
	MECPP	0.017	0.627	0.038	0.289
	MBzP	−0.053	0.135	−0.050	0.164
	MCPP	−0.004	0.915	0.025	0.493
	MEP	0.011	0.762	0.020	0.586
	MMP	0.080	0.024	0.094	0.009
Environmental phenols ^d^	BPA	0.078	0.028	0.082	0.023
	BPF	0.025	0.490	0.022	0.547
	BPS	0.098	0.006	0.098	0.007
	TCS	−0.032	0.375	−0.022	0.537
	MP	0.002	0.951	0.024	0.515
	EP	0.034	0.347	0.031	0.387
	PP	−0.019	0.594	−0.001	0.979
	BP	0.068	0.055	0.083	0.022
	BP_3	−0.012	0.744	0.004	0.905
VOCs ^e^	t,t-MA	0.116	0.001	0.126	0.001
	BMA	−0.019	0.596	0.008	0.833
PFAS ^f^	PFOA	−0.091	0.011	−0.091	0.012
	PFOS	−0.103	0.004	−0.114	0.002
	PFHxS	−0.076	0.032	−0.076	0.034
	PFNA	−0.073	0.041	−0.084	0.020
	PFDeA	−0.079	0.027	−0.084	0.019
Pesticide ^g^	PBA	0.055	0.126	0.052	0.151

* *r* (correlation coeafficient) and *p*-value were calculated using Pearson’s correlation. ** Adjusted by age, gender, BMI, household income, and alcohol drinking. ^a^ Heavy metal: blood lead (BPb), blood mercury (BHg), urine mercury (Uhg), and urine cadmium (Ucd). ^b^ PAHs (polycyclic aromatic hydrocarbons): 1-hydroxypyrene(OHP), 2-naphthol(NAP), 2-hydroxyfluorene (OHFlu), and 1-hydroxyphenanthrene (OHPhe). ^c^ Phthalates: mono-(2-ethyl-5-hydroxyhexyl) phthalate (MEHHP), mono-(2-ethyl-5-oxohexyl) phthalate (MEOHP), mono-n-butyl phthalate (MnBP), mono-(2-ethyl-5-carboxypentyl) phthalate (MECPP), monobenzyl phthalate (MBzP), mono(3-carboxypropyl) phthalate (MCPP), monoethyl phthalate (MEP), and monomethyl phthalate (MMP). ^d^ Environmental phenols: bisphenol A (BPA), bisphenol F (BPF), bisphenol S (BPS), triclosan (TCS), methyl paraben (MP), ethyl paraben (EP), propyl paraben (PP), butyl paraben (BP), and benzophenone-3 (BP_3). ^e^ VOCs: trans,trans-muconic acid (t,t-MA) and benzylmercapturic acid(BMA). ^f^ PFASs (per- and polyfluoroalkyl substances): perfluorooctanoic acid (PFOA), perfluorooctanesulfonate (PFOS), perfluorohexanesulfonic acid (PFHxS), perfluorononanoic acid (PFNA), and perfluorodecanoic acid (PFDeA). ^g^ Pesticide: 3-phenoxybenzoic acid (PBA).

## Data Availability

The data that support the findings of this study are available from the National Institute of Environmental Research, but restrictions apply to the availability of these data, which were used under license for the current study, and so are not publicly available. Data are, however, available from the authors upon reasonable request and with permission of the National Institute of Environmental Research.

## Supplementary Materials

The following supporting information can be downloaded at: https://www.mdpi.com/article/10.3390/toxics13070546/s1. Figure S1: Distribution trends of heavy metal levels by urinary cotinine levels; Figure S2: Distribution trends of PAHs levels by urinary cotinine levels, Figure S3: Distribution trends of phthalates levels by urinary cotinine levels, Figure S4: Distribution trends of environmental phenols levels by urinary cotinine levels, Figure S5: Distribution trends of VOCs levels by urinary cotinine levels, Figure S6: Distribution trends of PFAS levels by urinary cotinine levels, Figure S7: Distribution trends of pesticide levels by urinary cotinine levels, S8: Environmental Chemical Analysis Conditions, QA/QC Procedures, and Limits of Detection.

## Abbreviations

BMA: benzyl mercapturic acid; BP: butyl paraben; BP_3: benzophenone-3; BPb: blood lead; BHg: blood mercury; BPA: bisphenol a; BPF: bisphenol f; BPS: bisphenol s; COT: cotinine; DEHP: di-2-ethylhexyl phthalate; EP: ethyl paraben; ETS: environmental tobacco smoke; KoNEHS: Korean national environmental health survey; MBzP: monobenzyl phthalate; MECPP: mono-(2-ethyl-5-carboxypentyl) phthalate; MEHHP: mono-(2-ethyl-5-hydroxyhexyl) phthalate; MEOHP: mono-(2-ethyl-5-oxohexyl) phthalate; MEP: monoethyl phthalate; MMP: monomethyl phthalate; MnBP: mono-n-butyl phthalate; MP: methyl paraben; NAP: 2-naphthol; OHP: 1-hydroxypyrene; OHPhe: 1-hydroxyphenanthrene; OHFlu: 2-hydroxyfluorene; PAHs: polycyclic aromatic hydrocarbons; PBA: 3-phenoxybenzoic acid; PFASs: per- and polyfluoroalkyl substances; PFDeA: perfluorodecanoic acid; PFHxS: perfluorohexanesulfonic acid; PFNA: perfluorononanoic acid; PFOA: perfluorooctanoic acid; PFOS: perfluorooctanesulfonate; PP: propyl paraben; TCS: triclosan; t,t-MA: trans,trans-muconic acid; Ucd: urine cadmium; Uhg: urine mercury; and VOCs: volatile organic compounds.
