# Supplementary material for: Exposure to Environmental Chemicals from Environmental Tobacco Smoking in Korean Adolescents
Source: Toxics. 2025 Jun 29;13(7):546. doi: 10.3390/toxics13070546 (PMC12300141; doi:10.3390/toxics13070546)
Supplement: Supplementary file 1 [file toxics-13-00546-s001.zip › toxics-3724065-supplementary.pdf]

Supplementary legend

Figure S1. Distribution trends of heavy metal levels by urinary cotinine levels

All data were log-transformed.
Heavy metals: blood lead (BPb), blood mercury (BHg), urine mercury (Uhg), and urine cadmium (Ucd)

Figure S2. Distribution trends of PAHs levels by urinary cotinine levels

All data were log-transformed
PAHs (polycyclic aromatic hydrocarbons): 1-hydroxypyrene (OHP), and 1-hydroxyphenanthrene (OHPhe)

Figure S3. Distribution trends of phthalates levels by urinary cotinine levels

All data were log-transformed
Phthalates: mono-(2-ethyl-5-hydroxyhexyl) phthalate (MEHHP), mono-(2-ethyl-5-oxohexyl) phthalate (MEOHP), mono-n-butyl phthalate (MnBP), mono-(2-ethyl-5-carboxypentyl) phthalate (MECPP), monobenzyl phthalate (MBzP), mono(3-carboxypropyl) phthalate (MCPP), monoethyl phthalate (MEP), and monomethyl phthalate (MMP)

Figure S4. Distribution trends of environmental phenols levels by urinary cotinine levels

All data were log-transformed
Environmental phenols: bisphenol F (BPF), bisphenol S (BPS), triclosan (TCS), methyl paraben (MP), ethyl paraben (EP), propyl paraben (PP), butyl paraben (BP), and benzophenone-3 (BP_3)

Figure S5. Distribution trends of VOCs levels by urinary cotinine levels

All data were log-transformed
VOCs (volatile organic compounds): benzylmercapturic acid (BMA)

Figure S6. Distribution trends of PFAS levels by urinary cotinine levels

All data were log-transformed
PFAS (Per- and polyfluoroalkyl substances): perfluorooctanoic acid (PFOA), perfluorooctanesulfonate (PFOS), perfluorohexanesulfonic acid (PFHxS), perfluorononanoic acid (PFNA), and perfluorodecanoic acid (PFDeA)

Figure S7. Distribution trends of pesticide levels by urinary cotinine levels

All data were log-transformed
Pesticide: 3-phenoxybenzoic acid (PBA)

S8. Environmental Chemical Analysis Conditions, QA/QC Procedures, and Limits of Detection

Figure S1. Distribution trends of heavy metal levels by urinary cotinine levels

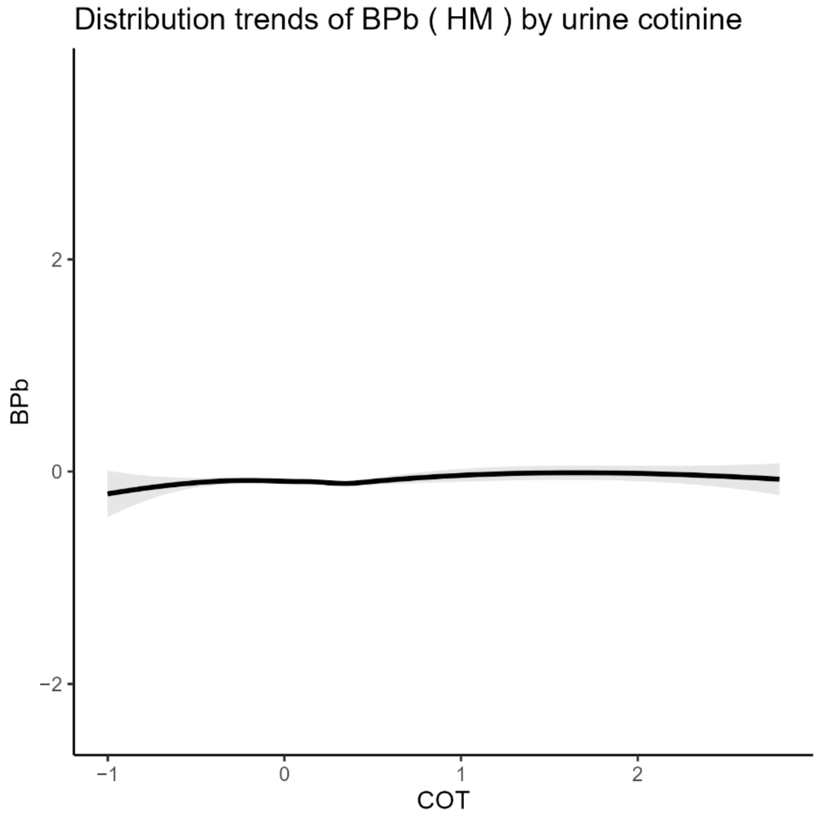

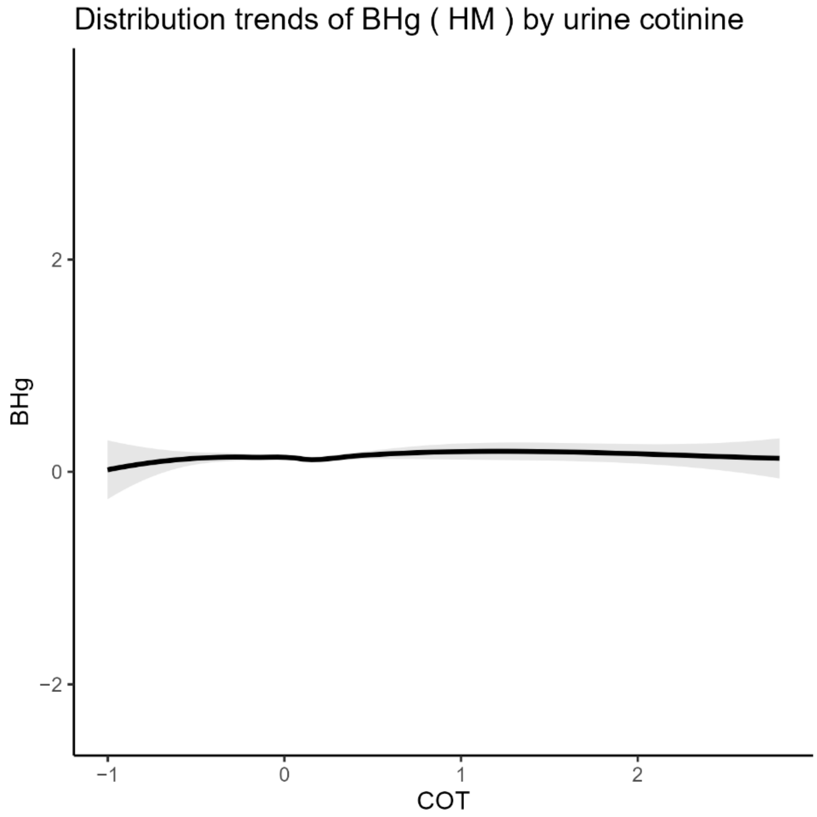

Distribution trends of Uhg (HM) by urine cotinine

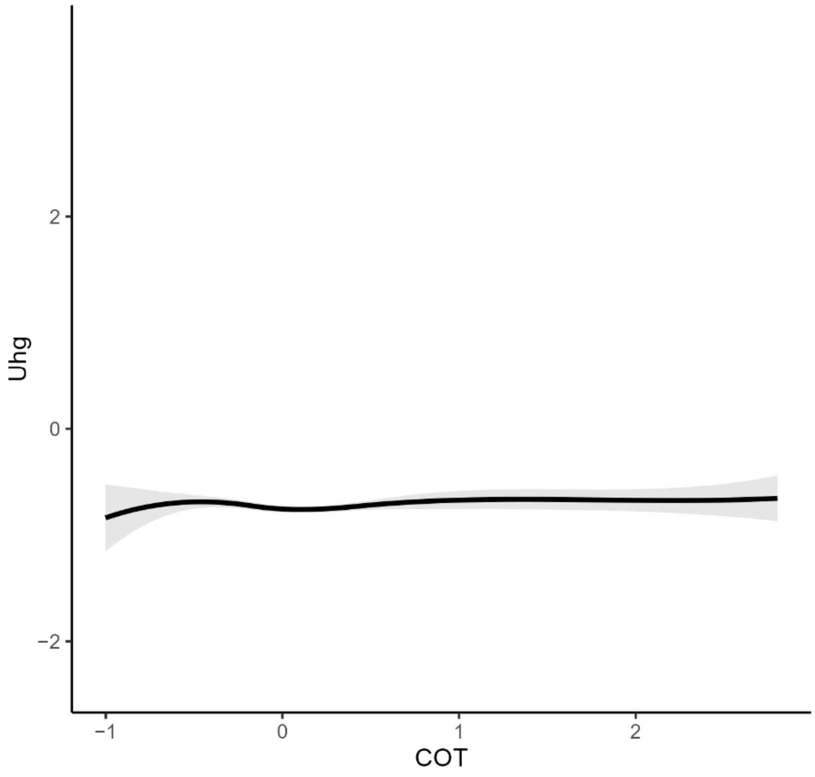

Distribution trends of Ucd (HM) by urine cotinine

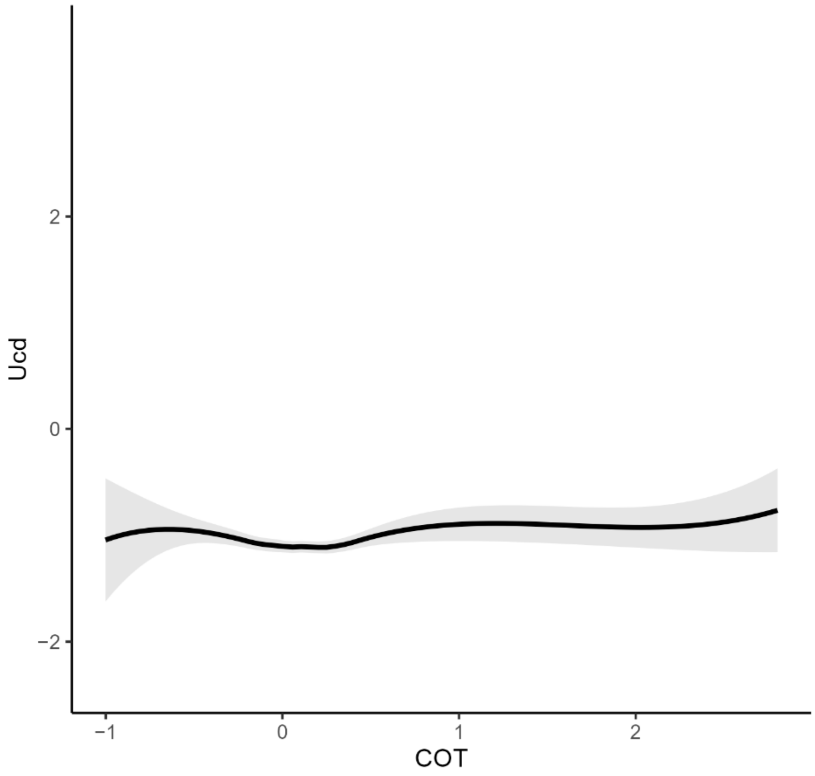

Figure S2. Distribution trends of PAHs levels by urinary cotinine levels

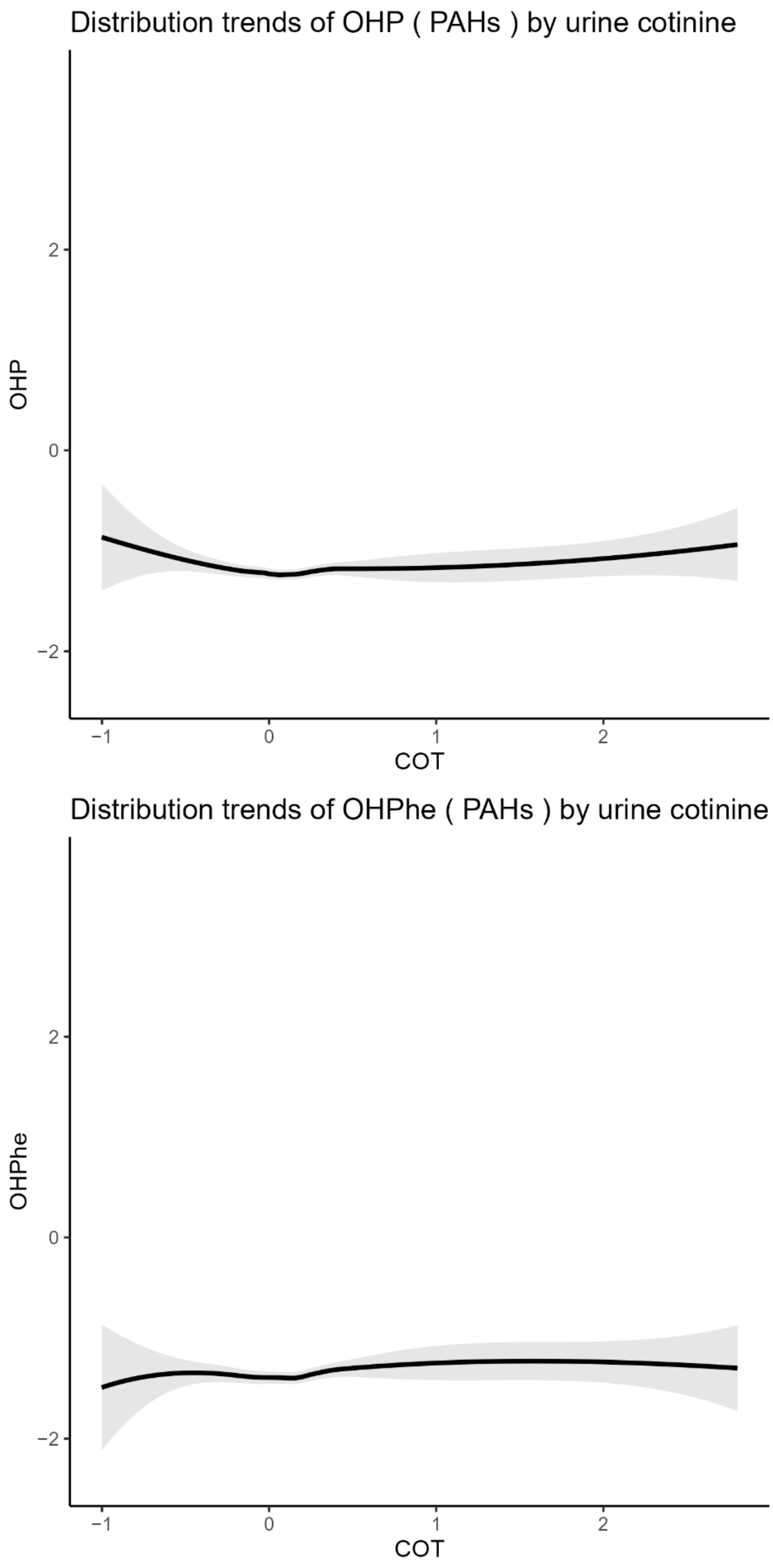

Figure S3. Distribution trends of phthalates levels by urinary cotinine levels

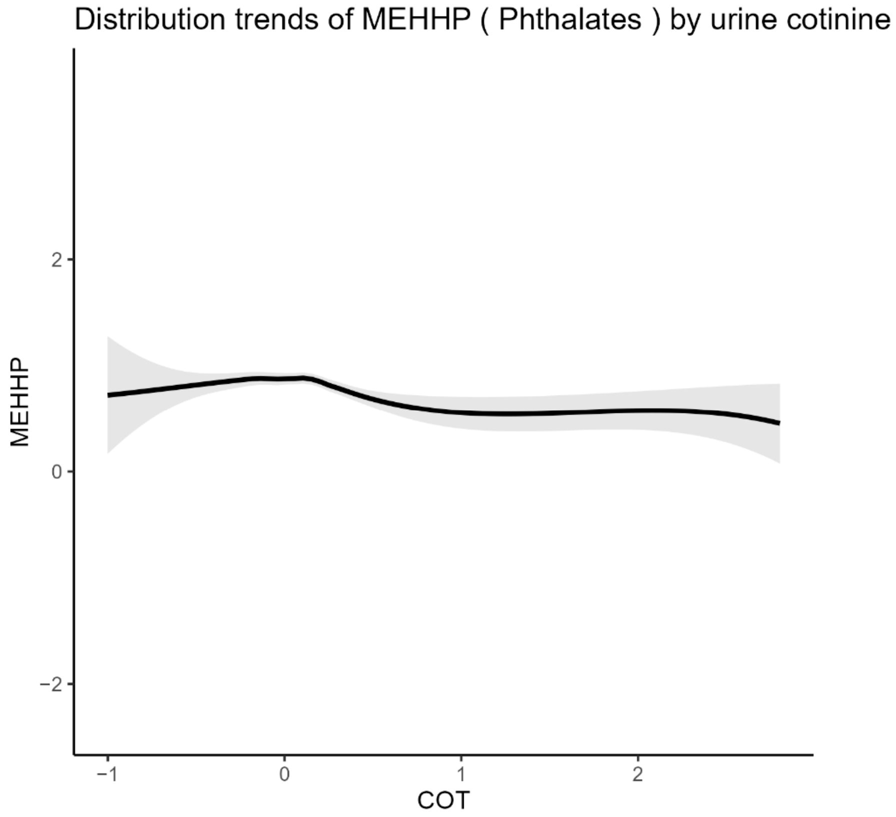

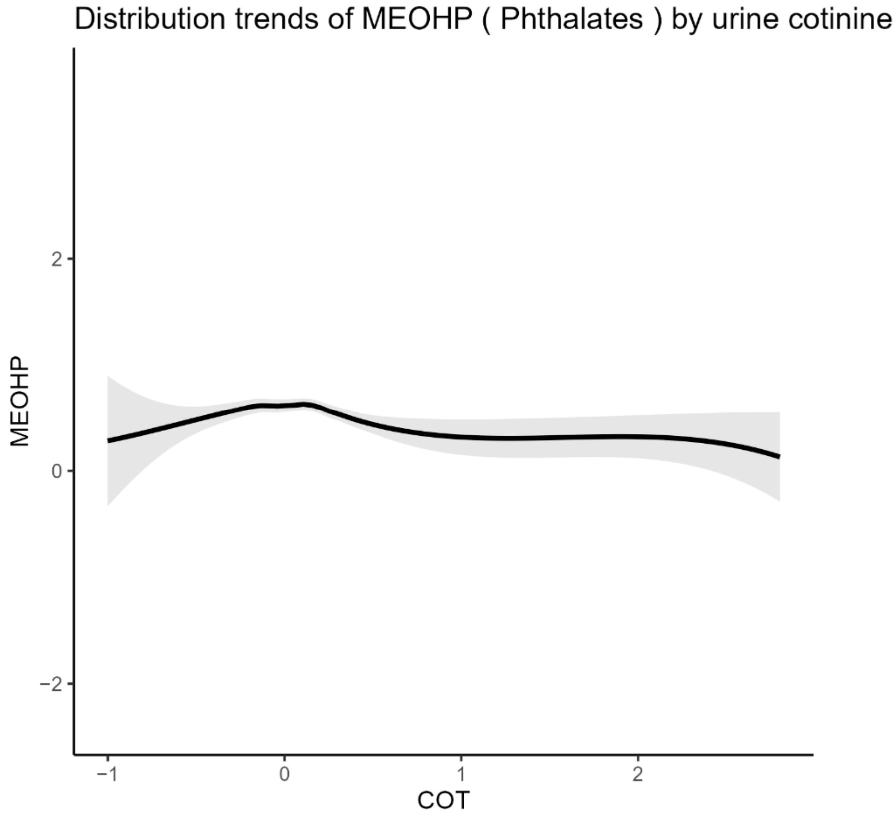

Distribution trends of MnBP (Phthalates) by urine cotinine

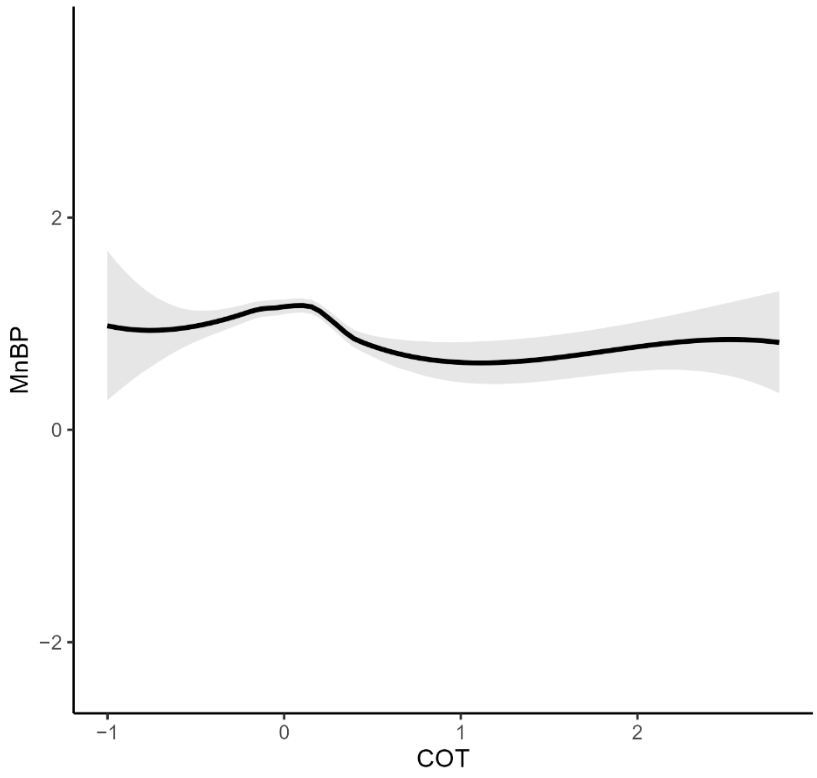

Distribution trends of MECPP (Phthalates) by urine cotinine

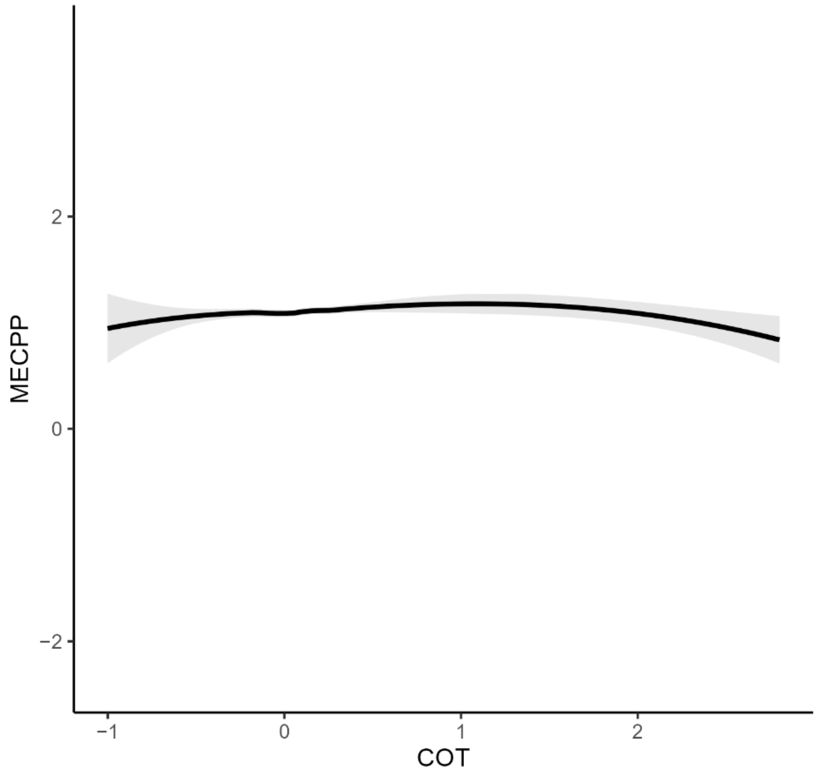

Distribution trends of MBzP (Phthalates) by urine cotinine

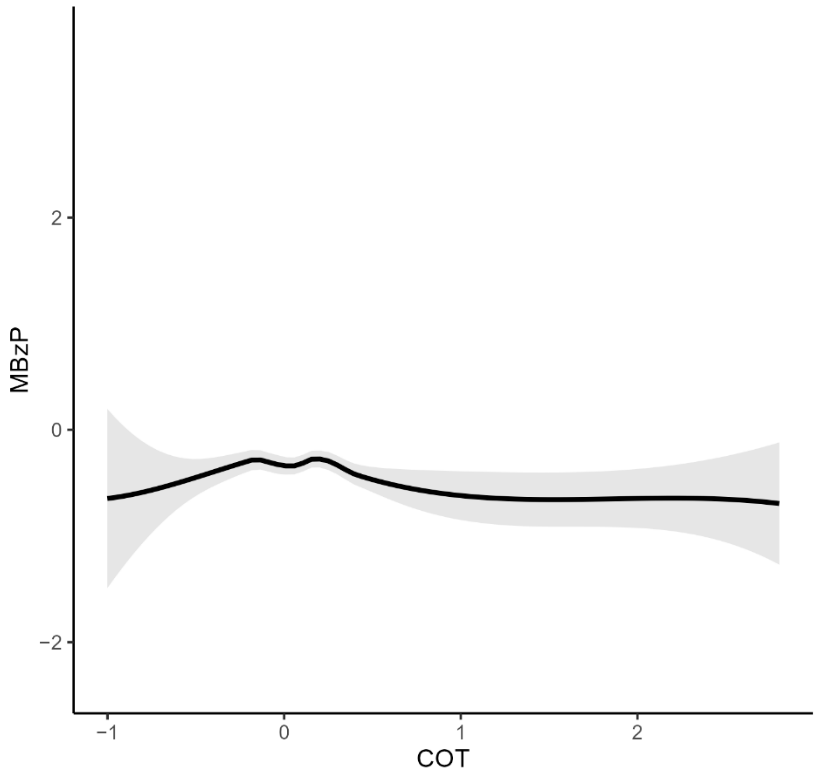

Distribution trends of MCPP (Phthalates) by urine cotinine

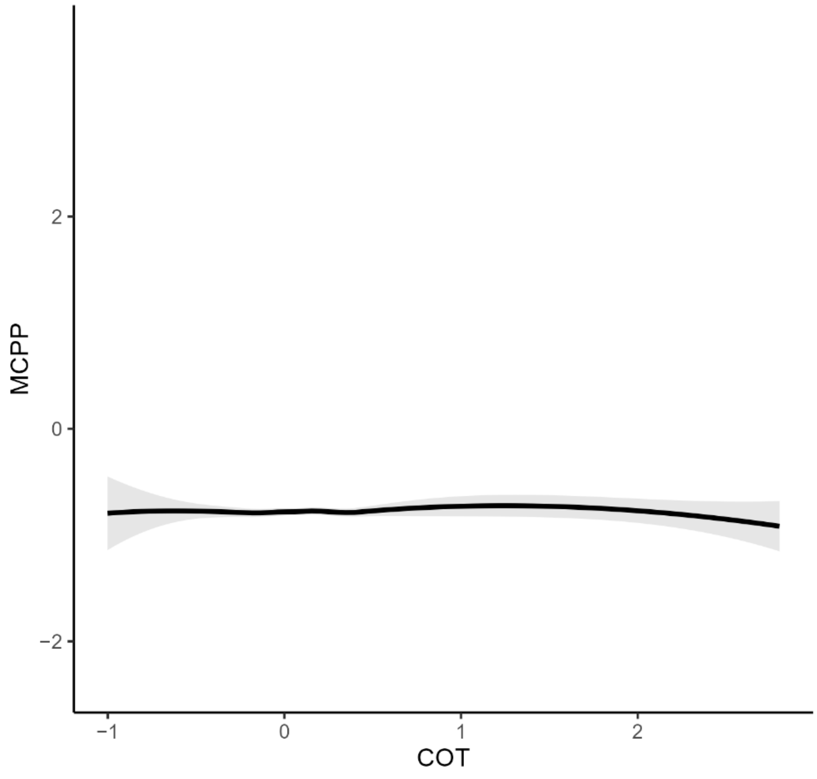

Distribution trends of MEP (Phthalates) by urine cotinine

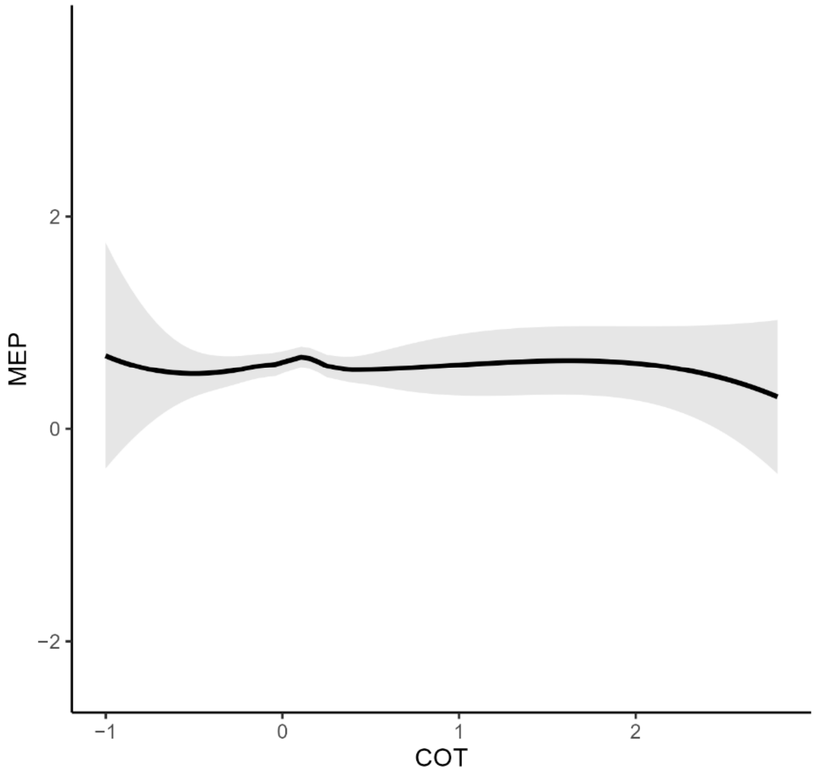

Distribution trends of MMP (Phthalates) by urine cotinine

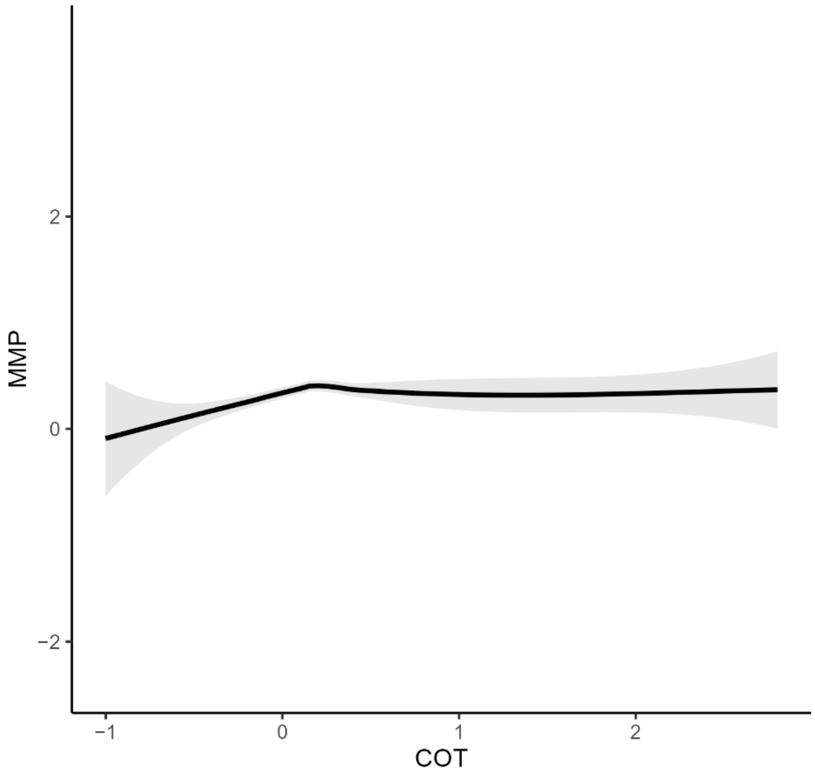

Figure S4. Distribution trends of environmental phenols levels by urinary cotinine levels

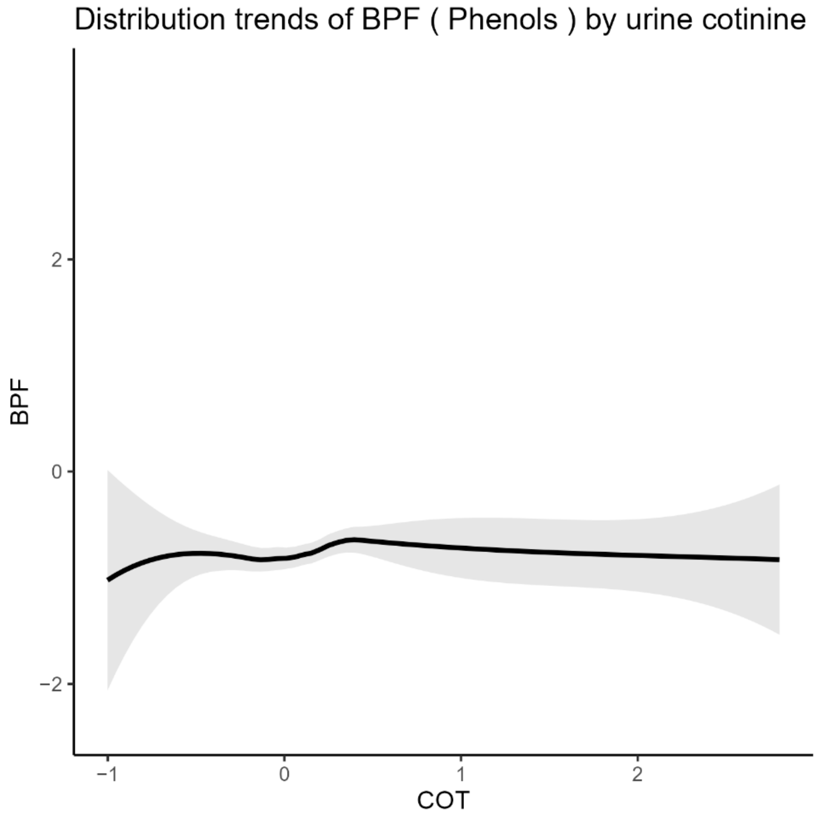

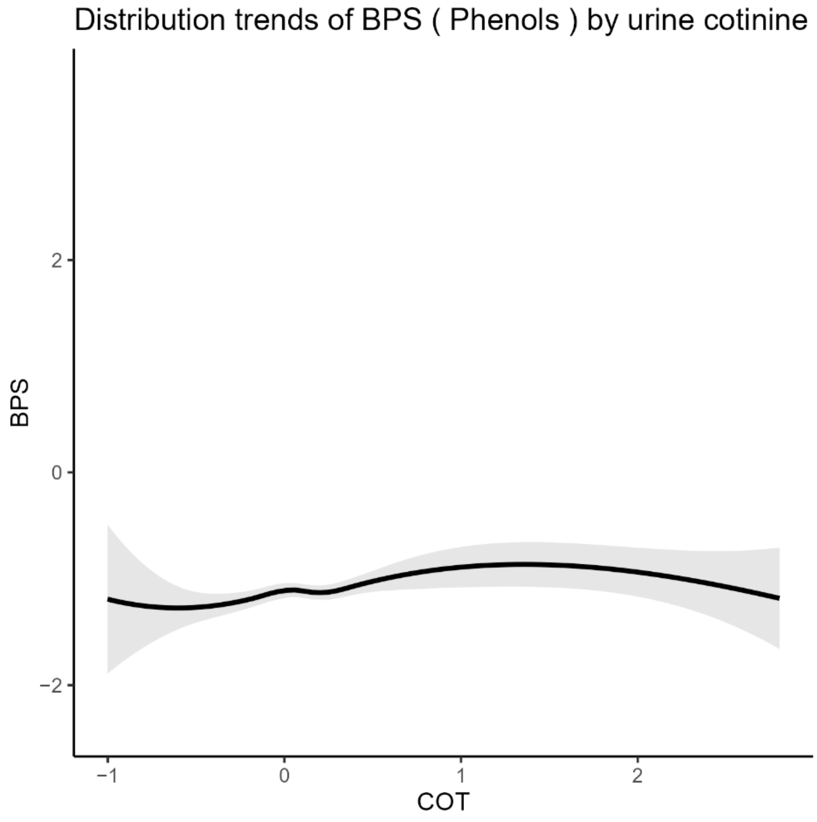

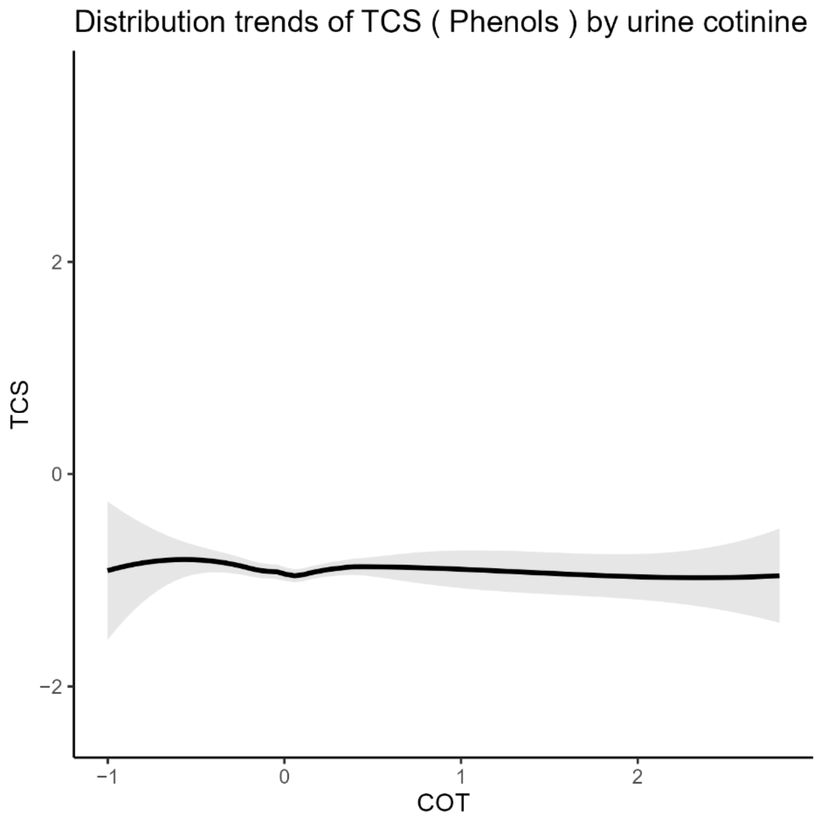
Distribution trends of TCS (Phenols) by urine cotinine
TCS
COT
2
0
−2
−1
0
1
2

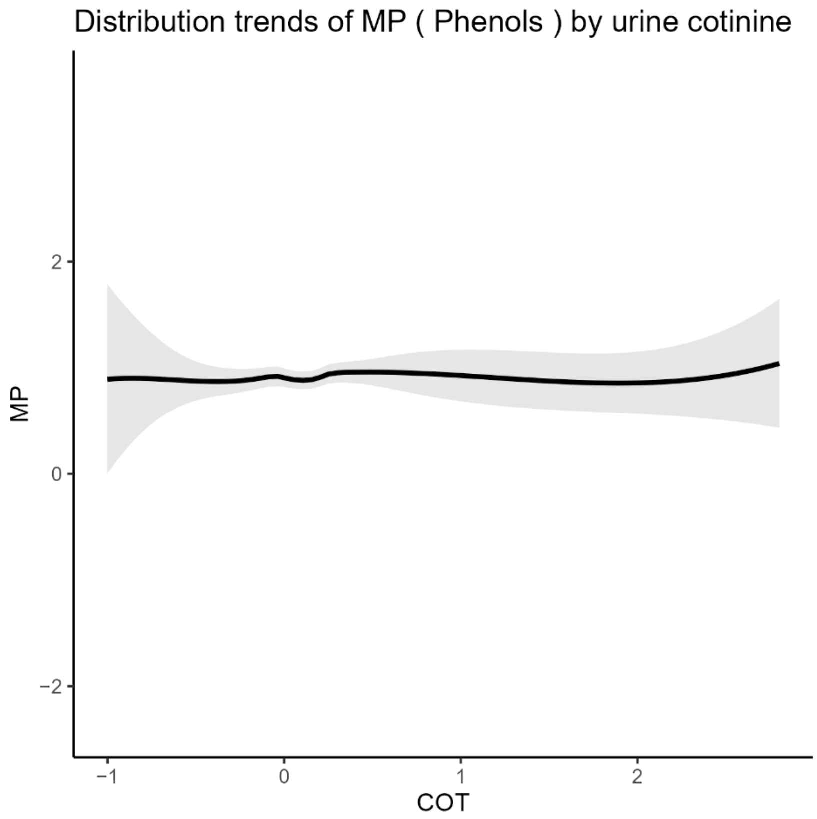
Distribution trends of MP (Phenols) by urine cotinine
MP
COT
2
0
−2
−1
0
1
2

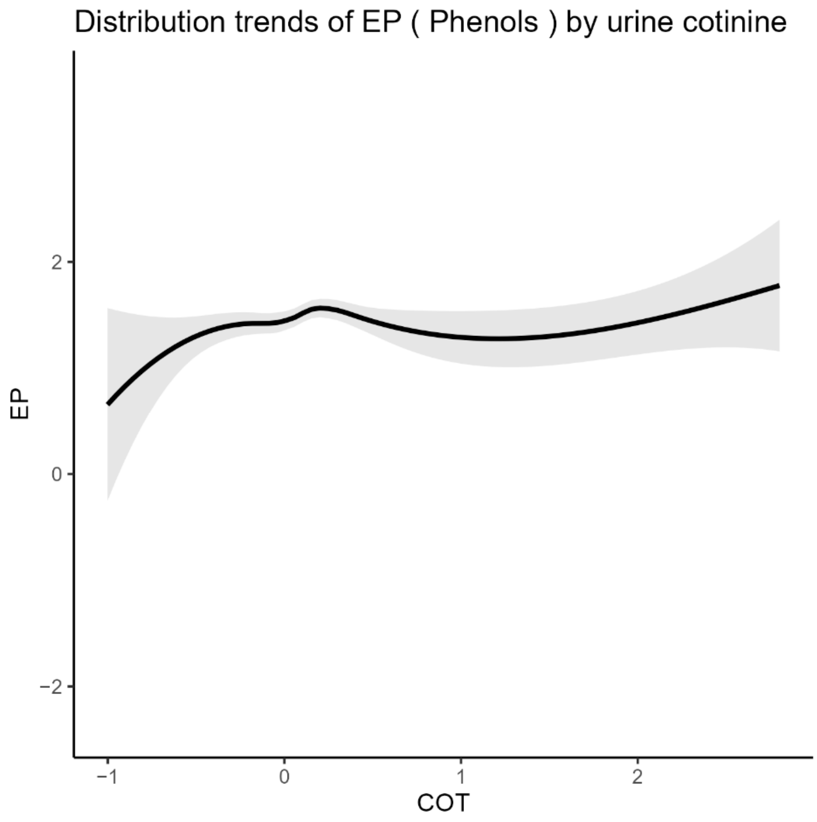
Distribution trends of EP (Phenols) by urine cotinine
EP
2
0
−2
−1
0
1
2
COT

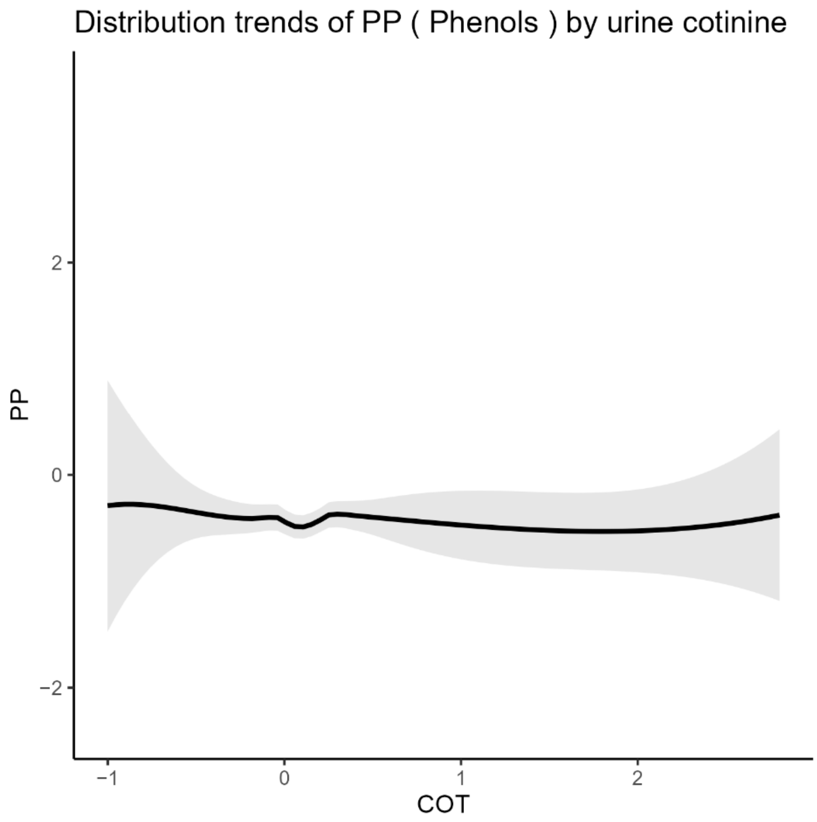
Distribution trends of PP (Phenols) by urine cotinine
PP
2
0
−2
−1
0
1
2
COT

Distribution trends of BP (Phenols) by urine cotinine

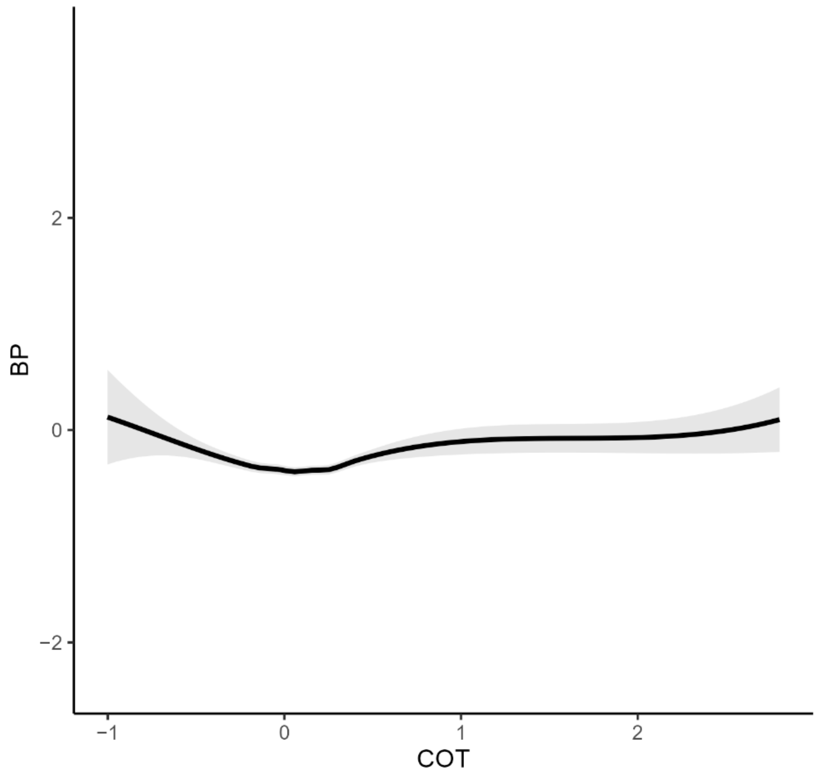

Distribution trends of BP_3 (Phenols) by urine cotinine

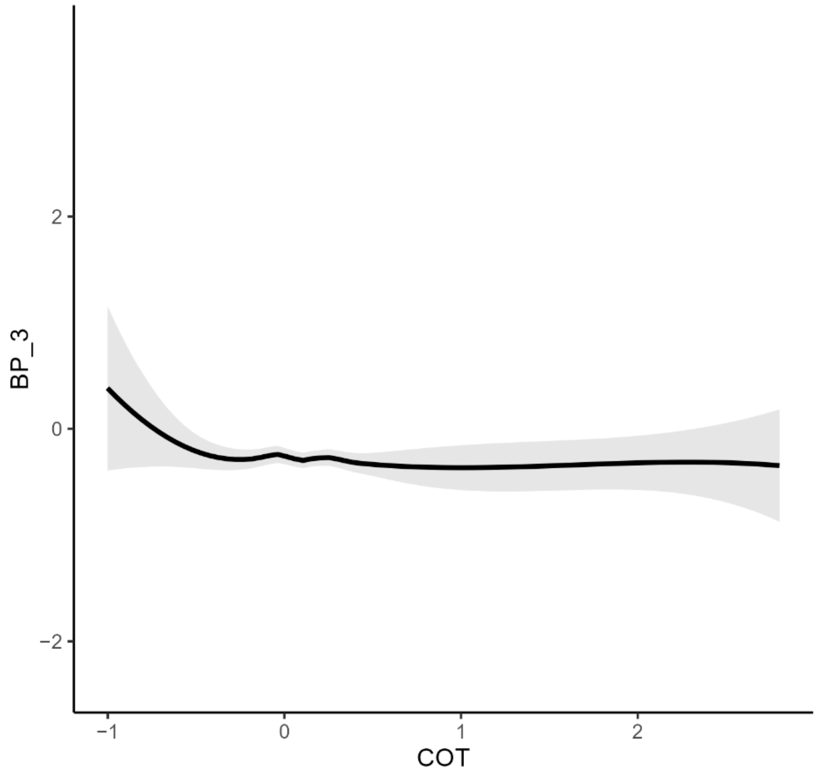

Figure S5. Distribution trends of VOCs levels by urinary cotinine levels

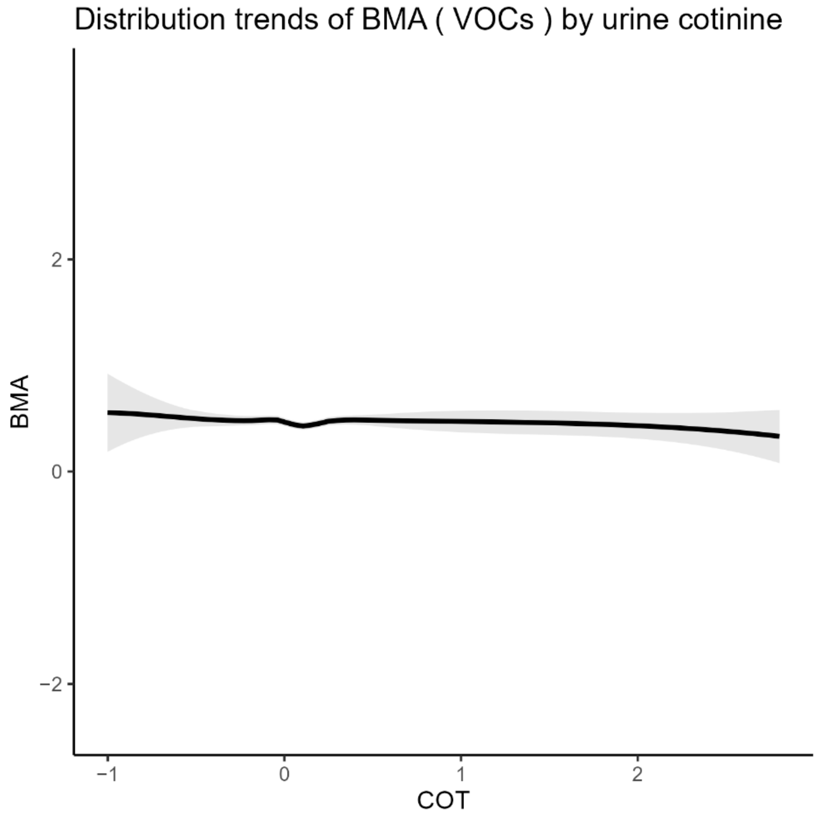

Figure S6. Distribution trends of PFAS levels by urinary cotinine levels

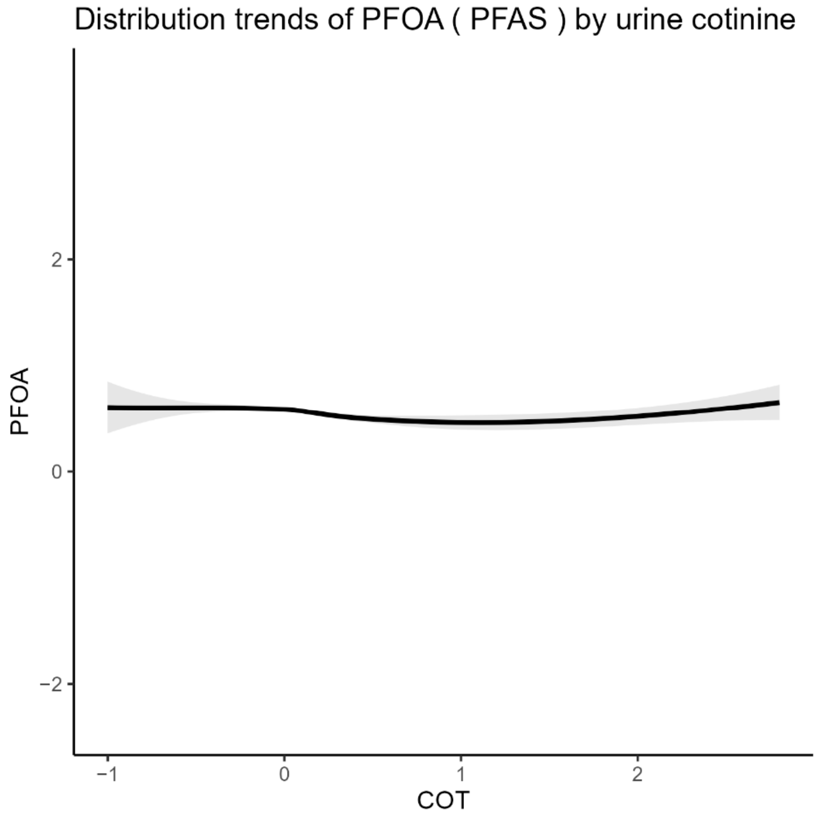

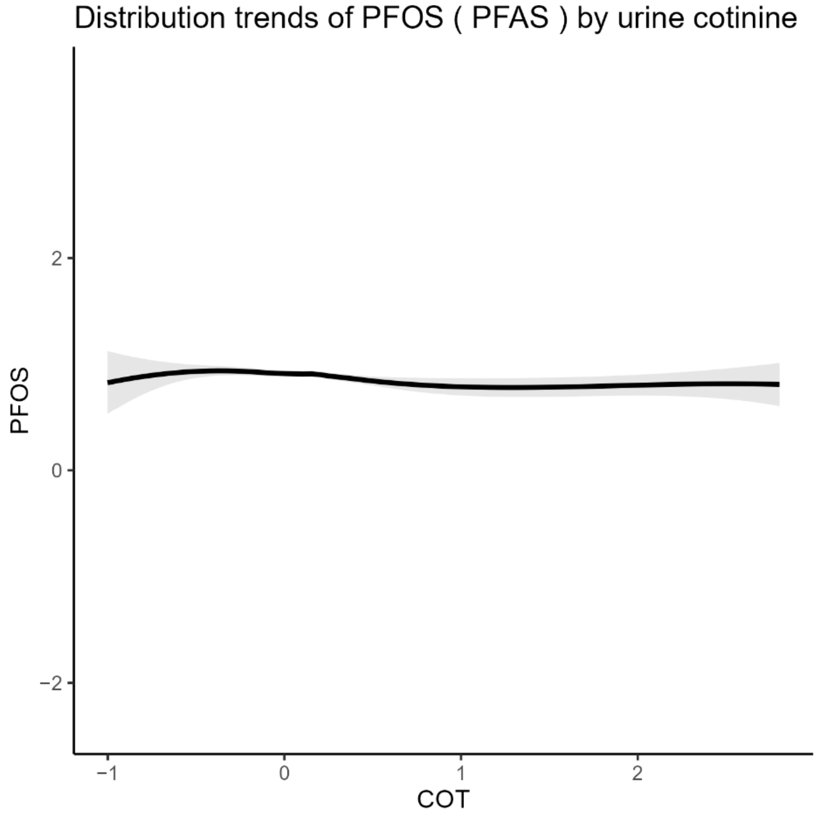

Distribution trends of PFHxS (PFAS) by urine cotinine

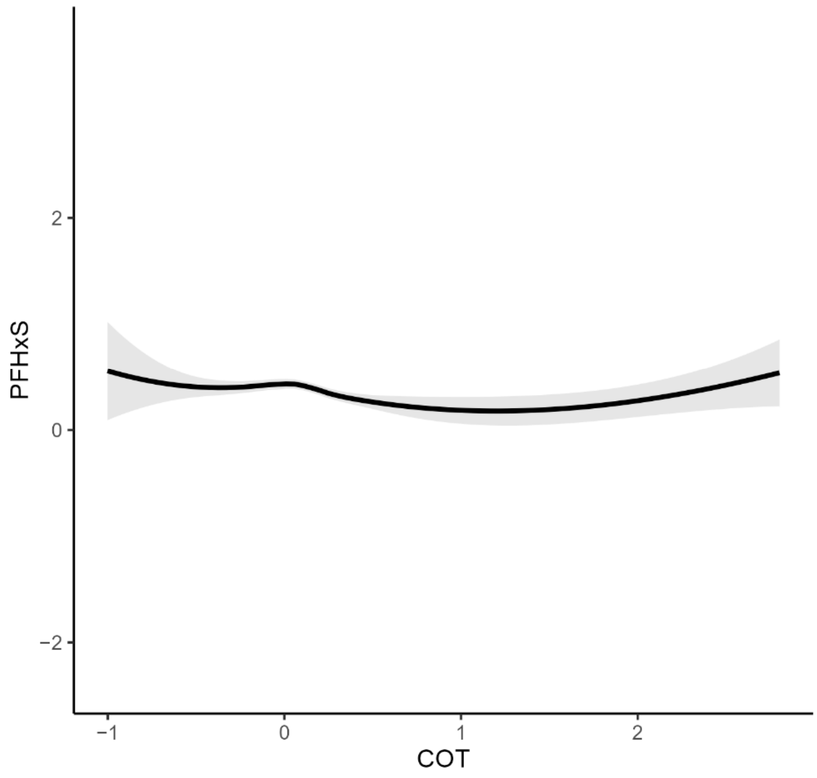

Distribution trends of PFNA (PFAS) by urine cotinine

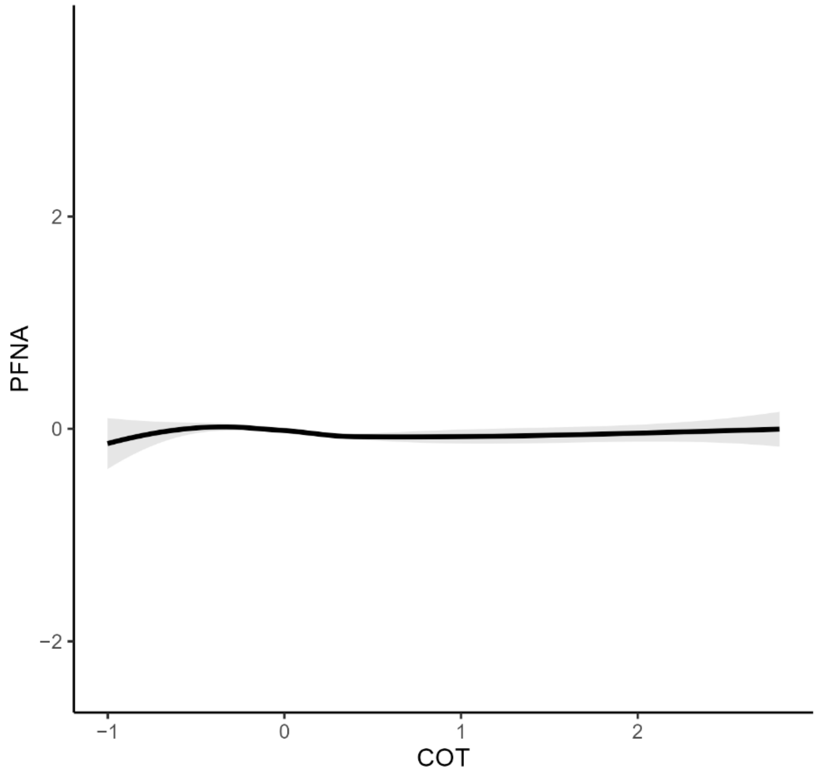

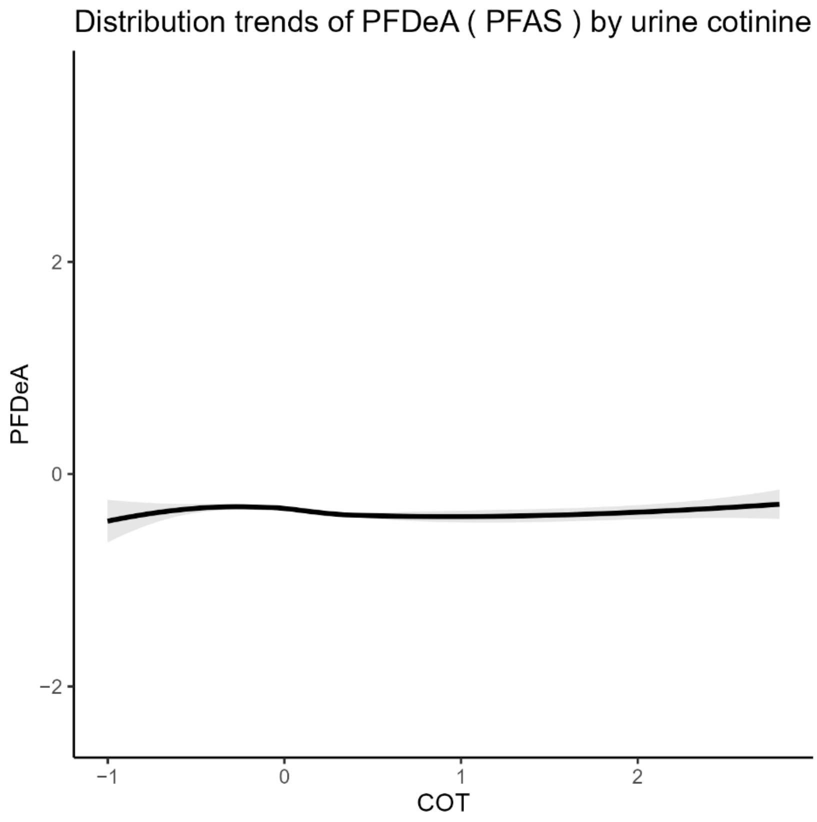
Distribution trends of PFDeA (PFAS) by urine cotinine
PFDeA
2
0
−2
−1
0
1
2
COT

Figure S7. Distribution trends of pesticide levels by urinary cotinine levels

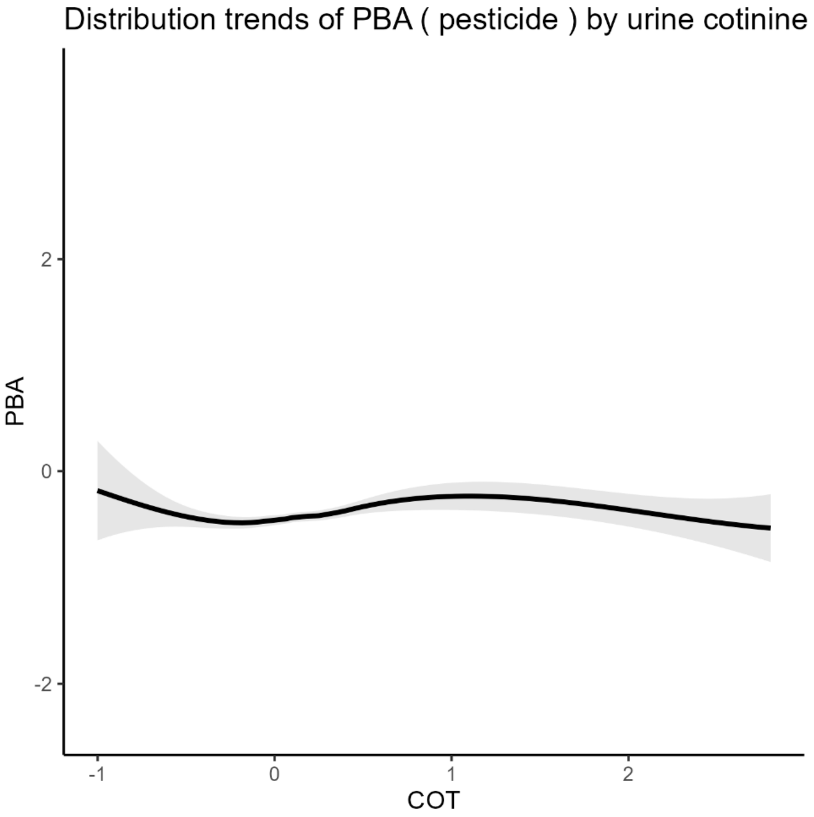

S8. Environmental Chemical Analysis Conditions, QA/QC Procedures, and Limits of Detection

1. Data source and sample collection

Data used in this study were obtained from Korean National Environmental Health Survey(KoNEHS), Cycle 4 (2018-2020). Blood samples were collected in containers and stored in a -70°C freezer. Urine samples were collected by each participant's guardian the day before or on the morning of the survey, and the collected urine samples were stored frozen at -20°C until analysis.

2. Analytical Methods and Instruments

Urine Cotinine: Urine Cotinine was analyzed using gas chromatography-mass spectrometry (GC-MS, Perkin Elmer).

Heavy metals: Blood lead (Pb) and urine cadmium (Cd) were analyzed using a graphite furnace atomic absorption spectrometer (GF-AAS, 900z, Perkin Elmer), and blood mercury (Hg) and urine mercury (Hg) were analyzed using a mercury-only analyzer (Gold amalgamation direct mercury analyzer, DMA-80, Milestone) using the gold amalgam method.

PAHs: A total of four PAH metabolites were analyzed in urine using gas chromatography-mass spectrometry (GC-MS, Perkin Elmer).

Phthalates: Analysis of phthalate metabolites in urine is quantitatively analyzed using HPLC-MS/MS (Agilent 6490/Agilent) in the negative mode of electron spray ionization (ESI), which has good selectivity due to the presence of hydroxyl groups.

Environmental Phenols: Analysis of environmental phenols in urine were quantitatively analyzed using HPLC-MS/MS (AB Sciex/API Triple Quad 5500).

VOCs: Analysis of VOCs in urine was quantitatively analyzed using HPLC-MS/MS (AB Sciex/API Triple Quad 5500).

PFAS: Serum PFAS were separated and quantified using a Q-sight Triple Quad high-performance liquid chromatography/mass spectrometer (PerkinElmer).

Pesticide: 3-PBA was analyzed using gas chromatography-mass spectrometry (GC-MS, Perkin Elmer).

3. Detection Limit

Limits of detection (LODs) for each metal were as follows:

Urine Cotinine (0.2 μg/L)

Heavy metals: blood Pb (0.17 μg/L), Blood Hg (0.1 μg/L), Urine Cd (0.04 μg/L), Urine Hg (0.04 μg/L)

PAHs: 1-OHP (0.044 μg/L), 1-OHPHE (0.037 μg/L), 2-NAP (0.033 μg/L), 2-OHFLU (0.052 μg/L)

Phthalates: MEHHP (0.069 μg/L), MEOHP (0.052 μg/L), MnBP (0.084 μg/L), MECPP (0.073 μg/L), MBzP (0.03 μg/L), MCPP (0.072 μg/L), MEP (0.108 μg/L), MMP (0.069 μg/L)

Environmental Phenols: BPA(0.029 μg/L), BPF(0.036 μg/L), BPS(0.022 μg/L), TCS(0.041 μg/L), MP(0.209 μg/L), EP(0.124 μg/L), PP(0.158 μg/L), BP(0.109 μg/L), BP-3(0.129 μg/L)

VOCs: t,t-MA(0.084 μg/L), BMA(0.136 μg/L)

PFAS: PFHxS (0.071 μg/L), PFOA (0.050 μg/L), PFNA (0.019 μg/L), PFOS (0.056 μg/L), and PFDeA (0.017 μg/L)

Pesticide: 3-PBA(0.02 μg/L)

4. Precision and Accuracy

To evaluate precision, duplicate analyses of quality control samples were performed (RM, CRM). Accuracy was verified using certified reference materials.

To check the sensitivity of the instrument, after the calibration curve is created, the calibration curve standard solution is measured every time 20 samples are analyzed. If it does not fall within 15% of the reference value, the previously analyzed 20 samples are analyzed by creating a calibration curve again.

5. Quality Control

Blank samples and spiked samples were analyzed in each batch.

Internal quality controls were performed for each run.

Participation in external quality assurance programs (G-EQUAS) confirmed inter-laboratory reliability twice a year.
